# Type I Clathrates as Novel Silicon Anodes: An Electrochemical and Structural Investigation

**DOI:** 10.1002/advs.201500057

**Published:** 2015-05-05

**Authors:** Ying Li, Rahul Raghavan, Nicholas A. Wagner, Stephen K. Davidowski, Loïc Baggetto, Ran Zhao, Qian Cheng, Jeffery L. Yarger, Gabriel M. Veith, Carol Ellis‐Terrell, Michael A. Miller, Kwai S. Chan, Candace K. Chan

**Affiliations:** ^1^ Materials Science and EngineeringSchool for Engineering of MatterTransport and EnergyArizona State UniversityTempeAZ85287USA; ^2^ Department of Chemistry and BiochemistryArizona State UniversityTempeAZ85287USA; ^3^ Materials Science and Technology DivisionOak Ridge National LaboratoryOak RidgeTN37831USA; ^4^ Department of Materials EngineeringSouthwest Research InstituteSan AntonioTX78238USA

**Keywords:** anode, Li‐ion battery, silicon, ternary type I clathrate

## Abstract

Silicon clathrates contain cage‐like structures that can encapsulate various guest atoms or molecules. An electrochemical evaluation of type I silicon clathrates based on Ba_8_Al*_y_*Si_46−*y*_ as the anode material for lithium‐ion batteries is presented here. Postcycling characterization with nuclear magnetic resonance and X‐ray diffraction shows no discernible structural or volume changes even after electrochemical insertion of 44 Li (≈1 Li/Si) into the clathrate structure. The observed properties are in stark contrast with lithiation of other silicon anodes, which become amorphous and suffer from large volume changes. The electrochemical reactions are proposed to occur as single phase reactions at approximately 0.2 and 0.4 V versus Li/Li^+^ during lithiation and delithiation, respectively, distinct from diamond cubic or amorphous silicon anodes. Reversible capacities as high as 499 mAh g−^1^ at a 5 mA g^−1^ rate were observed for silicon clathrate with composition Ba_8_Al_8.54_Si_37.46_, corresponding to ≈1.18 Li/Si. These results show that silicon clathrates could be promising durable anodes for lithium‐ion batteries.

## 1 Introduction

Due to their high charge storage capacities upon reaction with Li, diamond cubic^1^ (c‐Si) and amorphous silicon^2^ (a‐Si) are attractive anode materials for the development of high energy density lithium‐ion batteries. However, the 300% change in volume between the unlithiated and lithiated phases results in stress formation that can lead to pulverization of the silicon.^3^ A great deal of research has been devoted to better understand these phase transformations.^4^, ^5^, ^6^, ^7^ While nanostructured electrodes, such as those involving silicon nanowire,^8^ nanotube,^9^, ^10^, ^11^ nanocrystal,^12^ or nanoporous morphologies^13^, ^14^ have successfully demonstrated long‐term cycling without pulverization, this strategy relies on the use of engineered space within or between the silicon to allow free expansion and contraction. Agglomeration and degradation of the engineered structure after many lithiation/delithiation cycles may lower the effectiveness of this strategy. The current approach of nanostructuring also does not address the key fundamental problem with silicon, namely its large structural and volume changes upon lithiation. To this end, investigation of new anode materials with less drastic changes during reaction with Li is still required.

**Figure 1:**
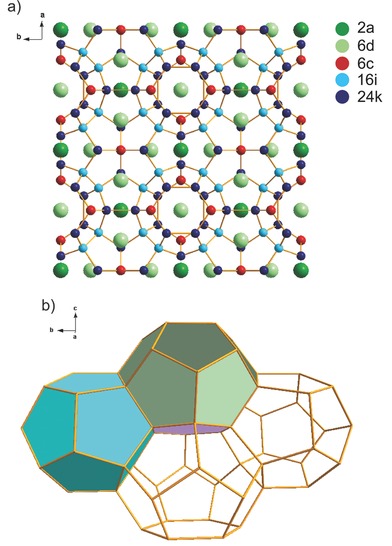
Structure of type I silicon clathrate. a) Ba_8_Si_46_ with crystallographic sites labeled using Wyckoff notation: Ba atoms are located at the 2a and 6d sites; Si atoms (and Al dopants) are at the 6c, 16i, and 24k sites. b) Polyhedral wire‐frame representation of the type I clathrate framework. Pentagonal dodecahedra (Si_20_ cages) are in light blue. Pentagonal faces of the tetrakaidecahedra (Si_24_ cages) are in green and the hexagonal face of the tetrakaidecahedra is in purple.

Silicon clathrates^15^ consist of silicon covalently bonded in face‐sharing Si_20_, Si_24_, and/or Si_28_ clusters with guest atoms occupying interstices inside the polyhedra. Their properties arise largely from their unique cage‐like structures and interactions between guest atoms with the clathrate framework. They are considered promising materials for superconducting^16^, ^17^, ^18^ and thermoelectric^19^, ^20^ applications. To date, their potential as energy storage materials has not been fully explored and their electrochemical properties are largely unknown. However, first principles calculations have pointed to the energetic feasibility of inserting Li into silicon clathrate structures with very little volume change.^21^, ^22^, ^23^ Type II clathrate based on Na_24_Si_136_ has been recently investigated as an anode for lithium‐ion batteries and was shown to become amorphous upon lithiation, similar to c‐Si.^23^, ^24^ Type II clathrates are composed of 16 pentagonal dodecahedra (Si_20_ cages) plus eight hexakaidecahedra (Si_28_ cages) per unit cell. In contrast, type I clathrates based on M*_x_*Si_46_ are composed of two Si_20_ cages plus six tetrakaidecahedra (Si_24_ cages) per unit cell. The guest atoms, M, are located in the 6d (center of the Si_24_ cages) and 2a sites (center of the Si_20_ cages), as described with Wyckoff symmetry notation (**Figure** **1**). Although the type II structures contain the larger Si_28_ cages, they also have a higher fraction of the smaller Si_20_ cages, which could be more susceptible to structural distortion or collapse upon lithiation. To our knowledge, the electrochemical characteristics of type I clathrates based on M*_x_*Si_46_ have not been experimentally investigated.

Here, we report our experimental evaluation of the electrochemical properties of type I clathrates based on Ba_8_Si_46_‐doped with Al. The advantage of the Ba system is that the type II clathrate Ba_24_Si_136_ is not formed as a by‐product during synthesis of Ba_8_Si_46_.^25^ In contrast, it is difficult to obtain Na_8_Si_46_ as a pure phase, as the typical synthesis utilizing thermal decomposition of NaSi can result in a mixture of Na_8_Si_46_ and Na_24_Si_136_,^23^, ^26^, ^27^ which are difficult to separate due to their similar densities and chemical properties. Substitution of the Si framework with other elements such as Al can decrease the clathrate melting point and enable synthesis of type I Ba clathrate using direct melting rather than high pressure methods^20^, ^28^, ^29^ while maintaining the cage structures.

Ternary type I silicon clathrates based on Ba_8_Al*_y_*Si_46−*y*_ were prepared using thermal annealing or arc melting. The clathrate electrodes were characterized before and after cycling using nuclear magnetic resonance (NMR), X‐ray diffraction (XRD), X‐ray photoelectron spectroscopy (XPS), and scanning electron microscopy (SEM). Electrochemical and structural analysis using XRD and NMR show that reversible lithium intercalation can occur in the type I clathrate without discernible structural changes to the silicon framework, with no volume change after even 44 Li (≈1 Li/Si) are inserted into each unit cell. However, long‐term cycling and capacity retention is found to be limited, particularly when cycling the clathrate electrodes at low current densities. Testing in different electrolytes and postmortem SEM analysis point to the potential role of the solid electrolyte interphase (SEI) layer in the observed cycling behavior.

## 2 Results

### 2.1 Characterization of Synthesized Ternary Silicon Clathrates

Polycrystalline powders were synthesized using thermal annealing and two different arc‐melting methods (lab scale arc‐melting in Ar atmosphere and industrial arc‐melting under vacuum). XRD analysis confirmed the type I structure for materials prepared using these three methods (Figure S1, Supporting Information). The samples synthesized using thermal annealing (Figure S1a, Supporting Information) and arc‐melting in Ar atmosphere (Figure S1b, Supporting Information) both contained BaSi_2_ side product and unreacted c‐Si, likely the result of insufficient mixing of the reactants. These nonclathrate materials were successfully removed using treatment with HCl and NaOH. The XRD pattern of the vacuum arc‐melted material displayed pure phase clathrate (Figure S1c, Supporting Information) but was also treated to remove any potential amorphous or undetected side products.

Rietveld analysis of the XRD patterns gave lattice constants of 10.56 Å for the thermally annealed material, 10.51 Å for the arc‐melted material in Ar atmosphere and 10.49 Å for the arc‐melted material under vacuum (Figure S2, Supporting Information), which may indicate small variations in Ba occupancy or degree of framework substitution between the three samples.^16^, ^20^, ^30^ Based on other studies on the Ba_8_Al*_y_*Si_46 −*y*_ system, the lattice parameter increases with increasing Al content,^20^ suggesting that the arc‐melted samples had less framework substitution. SEM imaging of the clathrate powder after ball‐milling showed particle size of about 1–10 μm. All of the samples showed a wide particle size distribution, but Gaussian‐type curve fitting revealed peak values at 0.57, 0.34, and 0.41 μm for the thermally annealed, Ar arc‐melted, and vacuum arc‐melted samples, respectively (Figure S3, Supporting Information).

Compositional analysis using wavelength‐dispersive X‐ray spectroscopy (WDS) showed average compositions of Ba_8_Al_10.39(4)_Si_35.61(1)_, Ba_8_Al_9.31(2)_Si_36.69(4)_, and Ba_8_Al_8.54(1)_Si_37.46(3)_ for the thermally annealed, Ar arc‐melted and vacuum arc‐melted samples, respectively (Ba was fixed at 8). Rietveld analysis of the XRD patterns showed compositions of Ba_7.71_Al_10.20_Si_35.80_, Ba_7.73_Al_10.13_Si_35.87_, and Ba_7.87_Al_9.81_Si_36.19_ for the thermally annealed, Ar arc‐melted, and vacuum arc‐melted samples, respectively. Precise determination of the composition of ternary silicon clathrates is not straight‐forward. Due to the similar scattering power between Al and Si, it is difficult to distinguish them using XRD refinement.^31^ However, the Al contents obtained by WDS and XRD are consistent with the trends observed in the lattice constants. While Ba deficiency in type I clathrates has been reported,^16^, ^30^ it is not clear to what extent the compositions obtained by WDS represent the true Ba content of our samples. For purposes of understanding the electrochemical behavior of the synthesized materials, the compositions of the clathrate samples are assumed to be the average compositions obtained from WDS.

### 2.2 Electrochemical Measurements

**Figure 2:**
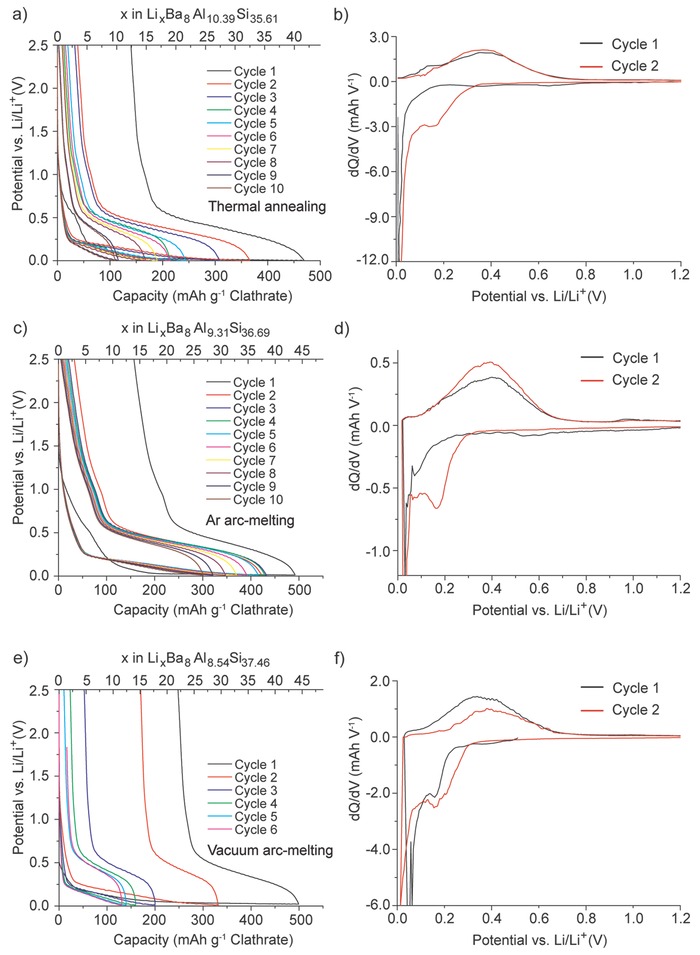
Galvanostatic cycling of clathrates in half‐cells using 5 mA g^−1^. a) Voltage profile and b) differential charge plots for thermally annealed samples. c) Voltage profile and d) differential charge plots for Ar arc‐melted samples. e) Voltage profile and f) differential charge plots for vacuum arc‐melted samples.

Electrochemical measurements of the clathrate electrodes in half‐cells with Li metal counter electrodes and electrolyte containing 1 m LiPF_6_ in ethylene carbonate (EC)/dimethyl carbonate (DMC)/diethyl carbonate (DEC) with vinylene carbonate (VC) additive indicated that the silicon clathrates were indeed electrochemically active. **Figure** **2** shows the voltage profile and differential charge (dQ/dV) plots for the clathrates using galvanostatic cycling at 5 mA g^−1^. The first lithiation (charging) curve for the clathrates showed a sloped profile displaying low reaction potentials (<50 mV vs Li/Li^+^) toward the latter half of the charge. The second cycles showed a slight decrease in capacity but with improved Coulombic efficiencies (CE) compared with those in the first cycles. Capacities of 469 mAh g^−1^ for thermally annealed clathrate (Figure 2a), 492 mAh g^−1^ for Ar arc‐melted clathrate (Figure 2c), and 499 mAh g^−1^ for vacuum arc‐melted clathrate (Figure 2e) were observed in the first charge based on the mass of the clathrate. This is equivalent to about 41, 43, and 44 Li inserted into each unit cell, respectively. The observed capacity of 499 mAh g^−1^ for vacuum arc‐melted Ba_8_Al_8.54_Si_37.46_ corresponds to 1129 mAh g^−1^ Si, if considering only the mass of the Si and excluding the mass Ba and Al, which is about one‐third of the theoretical lithiation capacity observed in c‐Si and a‐Si.^[1]^ The number of Li extracted in the first discharge was about 29, 30, and 22 for the thermally annealed, Ar arc‐melted, and vacuum arc‐melted clathrate, respectively.

From the dQ plots of the clathrate (Figure 2b,d,f), the potential‐dependent redox characteristics can be determined. The three different clathrate samples showed similar characteristics, with reduction starting below ≈0.2 V versus Li/Li^+^ in the first charge and ≈0.3 V in the second charge. The single broad peak observed in the dQ plots suggests a single‐phase, solid solution lithiation mechanism. A reduction feature at about 0.15 V versus Li/Li^+^ was observed in the first cycle of both arc‐melted samples, but not in the thermal‐annealed sample until the second cycle. The reason for this will require further study, but is likely due to the difference in composition of these three samples.

The electrochemical features observed for the three clathrates are very different from those observed in the lithiation of other silicon materials such as c‐Si,^1^, ^32^ a‐Si,^2^ SiO,^33^, ^34^ and polymer‐derived silicon ceramics.^35^, ^36^, ^37^, ^38^ c‐Si reacts with Li in a two‐phase process at 0.125 V versus Li/Li^+^ in the first charge to form amorphous lithium silicide (a‐Li*_x_*Si) and eventually transforms to crystalline Li_15_Si_4_ (c‐Li_15_Si_4_) at potentials below 50 mV versus Li/Li^+^. Lithiation of a‐Si occurs as two single‐phase reactions seen as broad peaks centered at 0.25 and 0.1 V versus Li/Li^+^ in dQ plots reflecting different Li–Si environments.^2^ SiO undergoes a conversion reaction with Li to form lithium silicates and Li_2_O; the first charge is characterized by a sharp peak in the differential charge plot at 0.25 V and a broad one at around 0.1 V versus Li/Li^+^. In contrast, the electrochemical lithiation features of the clathrates are much different. The differences are evident when comparing the dQ plots of the clathrates with those for c‐Si and a‐Si (Figure S4, Supporting Information). The clathrate reduction peaks are also different from those observed in polymer‐derived silicon ceramics such as SiCN,^35^, ^36^ which has major reduction peaks at around 0.3 V and 50 mV versus Li/Li^+^, or SiOC,^37^, ^38^ which undergoes lithiation over a broad voltage range starting at about 1 V versus Li/Li^+^.

The discharge characteristics of the clathrates are also very different from those observed during delithiation of c‐Li_15_Si_4_ and a‐Li*_x_*Si. c‐Li_15_Si_4_ undergoes a two‐phase delithiation reaction at about 0.43 V, while Li is removed from a‐Li*_x_*Si in two single‐phase reactions centered at 0.26 and 0.47 V versus Li/Li^+^ (Figure S4, Supporting Information). Delithiation of discharged SiO shows characteristics similar to delithiation of a‐Si.^33^ In the case of the clathrates, sloped voltage profiles were observed during discharge, and the dQ plots (Figure 2b,d,f) show a broad peak centered at 0.4 V versus Li/Li^+^ suggesting a single‐phase delithiation mechanism. From these data, we can see that the electrochemical properties of the clathrate are distinct and unique from those observed in lithiation of c‐Si, a‐Si, or SiO, or delithiation of c‐Li_15_Si_4_ or a‐Li*_x_*Si. This also excludes the possibility that the observed activity is due to residual c‐Si, a‐Si, or SiO present in the clathrate sample.

**Figure 3:**
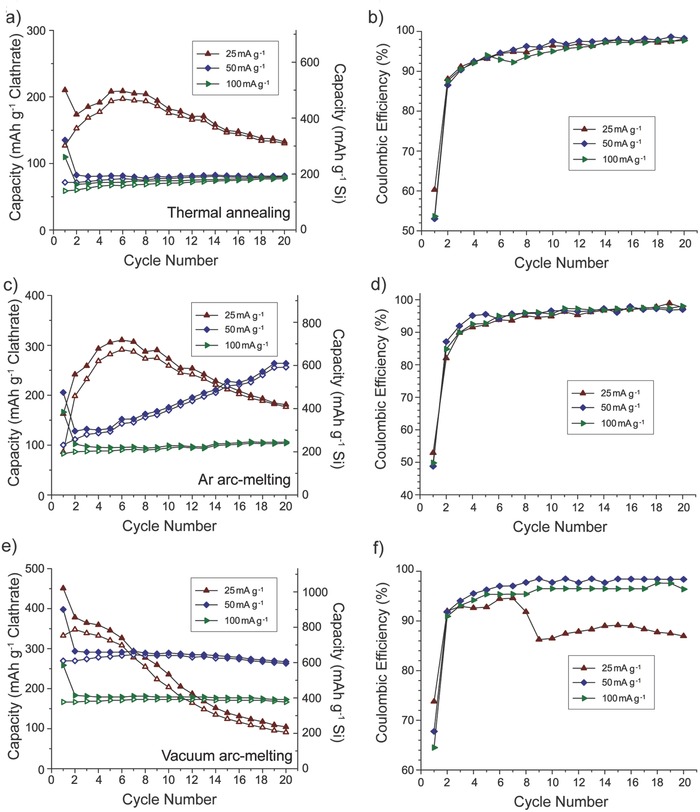
Galvanostatic cycling data for thermally annealed Ba_8_Al_10.39_Si_35.61_, Ar arc‐melted Ba_8_Al_9.31_Si_36.69_, and vacuum arc‐melted Ba_8_Al_8.54_Si_37.46_ using different current rates. a,c,e) Capacity versus cycle life. Capacity calculated using mass of entire clathrate (left axis) or mass of only Si (right axis). b,d,f) Coulombic efficiency versus cycle life.

Galvanostatic cycling of the clathrates was performed using different rates (25, 50, and 100 mA g^−1^) calculated based on the mass of the clathrate in the sample. **Figure** **3** shows the capacity and CE obtained for 20 cycles, while the voltage profiles can be found in Figure S5 (Supporting Information). The gravimetric capacities in Figure 3 were calculated with respect to the total mass of clathrate (left axis) and the mass of Si only (right axis). While the first cycle CE were low, in the range of 49–74%, the CE increased to 90–97% after 5 cycles. Figure 3a shows the cycling performance of thermally annealed clathrate. At the 20th cycle, the charge capacities were 133, 82, and 79 mAh g^−1^, for 25, 50, and 100 mA g^−1^, respectively. The corresponding capacity retention was 63%, 60%, and 72%, respectively. The Ar arc‐melted clathrate had low initial capacities at 25 and 50 mA g^−1^ but the capacity was observed to increase, reach a maximum, and then decrease. When the Ar arc‐melted clathrate was cycled at 25 mA g^−1^, the maximum capacity of 311 mAh g^−1^ was reached at the sixth cycle (Figure 3c); using 50 mA g^−1^, the maximum capacity of 314 mAh g^−1^ was reached at the 36th cycle (Figure S6, Supporting Information). The origin of the increase and decrease in capacity is still not known and will require further study. In general, the vacuum arc‐melted sample displayed higher specific capacities at all three current densities used (Figure 3e). The vacuum arc‐melted clathrates tested at 50 and 100 mA g^−1^ had lower capacities than at 25 mA g^−1^ but higher CE reaching 97–98% after 20 cycles (Figure 3f) and better capacity retention. Extended cycling using 25 and 100 mA g^−1^ also showed better capacity retention but lower capacities with the higher cycling rate (Figure S7a,b, Supporting Information).

From these data, we observe that despite the similar compositions and similar potential‐dependent redox behavior in the three samples, the clathrate preparation methods do appear to play a role on the cycling performance. The clathrate prepared using vacuum arc‐melting showed the highest specific capacities and best capacity retention at higher current densities. Unlike the other two samples, the vacuum arc‐melted clathrate did not contain side products after the synthesis, which implies a more homogenous mixing of the precursors. It should be noted that the specific capacities also increased as the Al content in the clathrate decreased, with the lowest Al content in the vacuum arc‐melted sample. The vacuum arc‐melted sample also had the highest Ba content from the XRD refinement results. However, control tests on half cells containing Li metal and Ba showed negligible reaction (Figure S8, Supporting Information). Hence, the significance of the composition trend, if any, will require more study.

### 2.3 NMR Characterization Before and After Lithiation

To better characterize the local environment of the silicon clathrate, static ^29^Si and ^7^Li NMR spectroscopy was performed on both fresh and chemically lithiated thermally annealed Ba_8_Al_10.39_Si_35.61_ and Ar arc‐melted Ba_8_Al_9.31_Si_36.69_ clathrates. Chemical lithiation of silicon anodes can be performed by placing the electrodes in direct contact with Li metal. Prior studies on prelithiation of silicon nanowires using this method^39^ showed that the structural transformations from chemical lithiation were very similar to those observed after electrochemical lithiation. Chemical lithiation with Li metal was used to avoid introduction of electrolyte and corresponding chemical shifts from the Li in the salt and SEI layer. Samples for NMR analysis were flame‐sealed in evacuated glass tubes to prevent oxidation by air. Electrochemical delithiation of identical, chemically lithiated Ar arc‐melted Ba_8_Al_9.31_Si_36.69_ electrodes showed that 26 Li were inserted into the clathrate electrodes under the prelithiation procedures used (Figure S9a, Supporting Information).

**Figure 4:**
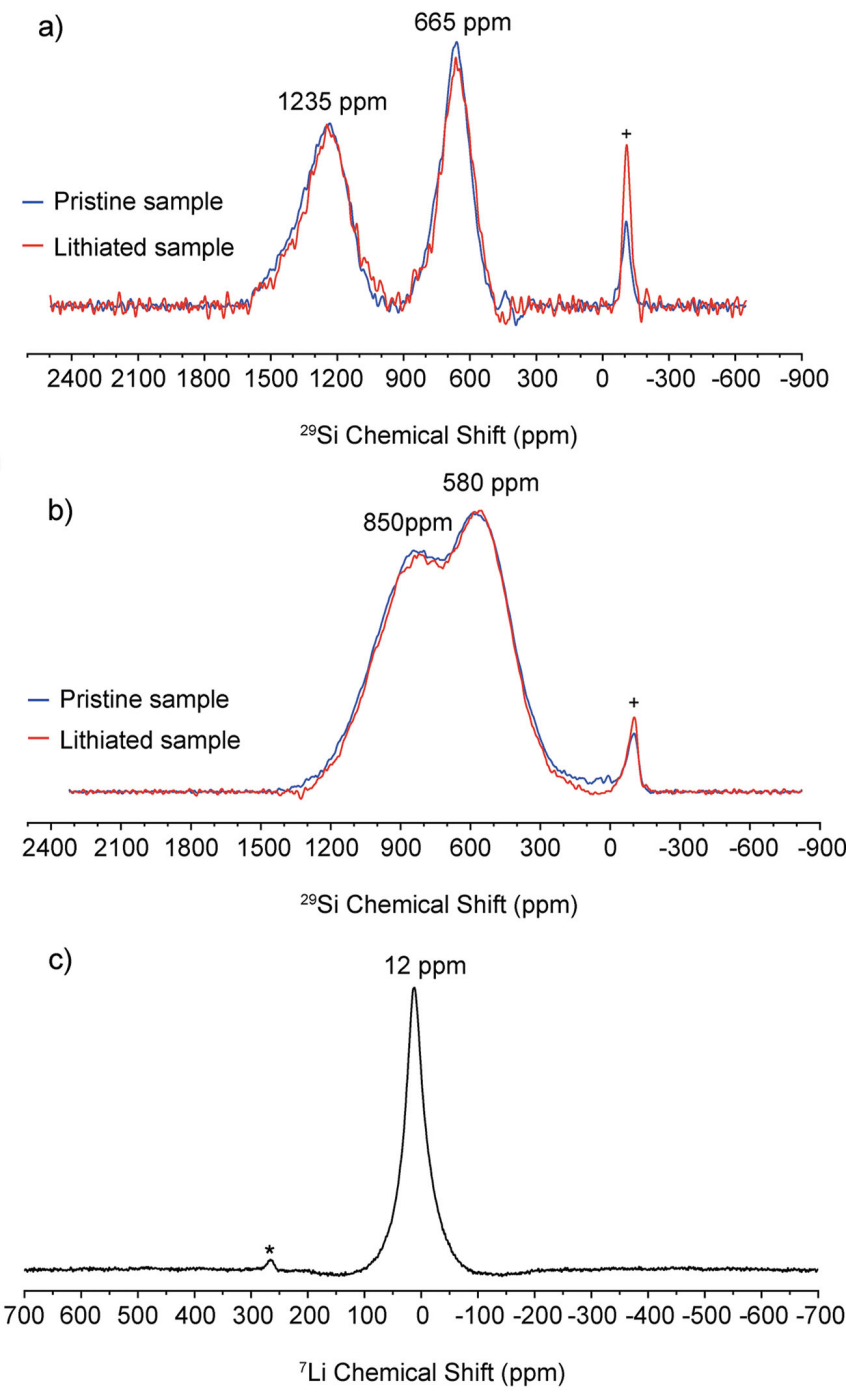
^29^Si static NMR spectra of a) thermally annealed Ba_8_Al_10.39_Si_35.61_ and b) Ar arc‐melted Ba_8_Al_9.31_Si_36.69_ (Peak labeled with “+” is from SiO_2_ glass tube used in static NMR). c) ^7^Li static NMR spectrum of Ar arc‐melted Ba_8_Al_9.31_Si_36.69_ (Peak labeled with an asterisk is from metallic lithium). The ^29^Si spectra were collected using the Hahn echo sequence. The ^7^Li static NMR spectra were obtained using a single pulse sequence.

**Figure** **4** shows the ^29^Si static NMR spectra of the thermally annealed and Ar arc‐melted clathrates before and after chemical lithiation. Two Knight‐shifted signals were observed at 665 and 1235 ppm for the thermal‐annealed clathrate (Figure 4a) with respect to the tetrakis(trimethylsilyl)silane (TTSS) reference compound. For the Ar arc‐melted clathrate (Figure 4b), the Knight shifted signals were centered at 580 and 850 ppm. NMR shifts for insulating and tetrahedrally coordinated Si compounds usually occur from 0 to −130 ppm, but large positive shifts are observed in silicon clathrates due to the contribution of electrons from the guest atoms to the conduction band.^26^, ^40^ Although the three crystallographic Si sites (Figure 1) can often be distinguished with ^29^Si NMR in unsubstituted type I clathrates,^26^, ^41^, ^42^, ^43^ the NMR spectra for ternary clathrates often show one or two broad peaks.^40^, ^44^, ^45^ The reason for the difference in ^29^Si chemical shifts between the thermally annealed and Ar arc‐melted clathrate is not yet understood. A similar observation was made in previous studies^44^ investigating Ba_8_Au*_y_*Si_46−*y*_, where the ^29^Si NMR spectra for *y* = 5.43 and 5.89 were very different from each other. These data may point to the origin of the differences in cycling performance observed between the clathrate samples. One possibility is that due to the differences in composition, and hence distribution of Al among the three crystallographic Si sites, the Li insertion/deinsertion processes are different as a result of the different local Si bonding environments, but this will require further study.

Notably, the ^29^Si NMR spectra of the pristine samples did not show resonances associated with c‐Si (expected at ca. −80 ppm) nor a‐Si (ca. −59 ppm),^4^, ^46^ which is consistent with our XRD data showing that unreacted Si could be removed with postsynthesis etching (Figure S1, Supporting Information) and further eliminating the possibility that the observed electrochemical reactions were due to residual c‐Si or a‐Si. More importantly, the NMR spectra of the chemically lithiated clathrates were very similar to those of the pristine clathrates, with the peak positions and line widths of each signal remaining unchanged after lithiation. This was further confirmed by conducting NMR spectroscopy with a longer (5 s) relaxation delay (Figure S9b, Supporting Information), which also showed no change between the pristine and lithiated clathrate. Furthermore, no resonances associated with Si that is part of an amorphous Li*_x_*Si phase or crystalline binary lithium silicide phase were observed. From previous studies of lithiated c‐Si, the ^29^Si NMR spectrum of the fully charged electrode is characterized by broad resonances at 72 and 235 ppm, thought to be associated with the formation of Li_15_Si_4_.^4^ The silicides with lower lithium content, Li_13_Si_4_ and Li_7_Si_3_, have Si chemical shifts between 301–342 ppm at room temperature.^47^ Also, the ^29^Si shift in LiSi is found at −106.5 ppm.^48^ The absence of peaks at these chemical shifts suggests that the clathrates do not undergo transformations to any of these lithium silicides. These results suggest that the clathrate structure was preserved and no amorphization occurred during the lithiation process.

To further gain insight into the lithiation mechanism, ^7^Li static NMR was performed on the Ar arc‐melted clathrate (Figure 4c). The spectrum shows one sharp peak centered at 12 ppm, which is close to the resonance found in Li_13_Si_4_ (11.5 ppm).^4^ Recent studies on the thermodynamic lithium silicide phases (e.g., Li_21_Si_5_, Li_13_Si_4_, Li_7_Si_3_, Li_12_Si_7_, and LiSi),^4^, ^47^ the metastable Li_15_Si_4_, as well as amorphous Li*_x_*Si formed from electrochemical lithiation of c‐Si^4^, ^49^, ^50^ have shown that the ^7^Li NMR chemical shifts associated with Li in silicon compounds is generally around 6–20 ppm. The overall trend among the lithium silicide phases is that the Li chemical shift decreases to lower frequencies as the Li:Si atomic ratio in the compound increases from 1.71 in Li_12_Si_7_ to 3.75 in Li_15_Si_4_, although Li_12_Si_7_ also shows an upshifted signal at −17.2 ppm, attributed to Li ions sandwiched between aromatic‐like Si_5_^6−^ rings.^51^, ^52^ Considering only the Li:Si ratio, the ^7^Li chemical shift observed in the lithiated clathrate does not follow the same trend, since the lithiated clathrate had Li:Si of approximately 0.71:1, as inferred from the discharge curve (Figure S9a, Supporting Information). The resonance is also not similar to what was observed after electrochemical insertion of Li into the empty sites of type II clathrate Si_136_, in which Knight‐shifted resonances at 412 and 433 ppm were attributed to a single Li atom in the Si_20_ and Si_28_ cages, respectively.^24^ The absence of Knight‐shifted ^7^Li resonances here could be because the silicon cages in our pristine materials are already filled by Ba, which introduces considerable electron density near the Fermi level.^53^

However, the resonance in our sample is similar to the chemical shift observed for LiSi at 12.6 ppm.^48^ The structure of LiSi is composed of 3‐coordinated Si arranged in puckered 8‐member rings with cavities 6–8 Å across, in which Li atoms reside in tetrahedral groupings and have bonds 2.6–3.1 Å to 6 Si neighbors.^54^ Although it is not exactly the same structural environment as the cages in the clathrate, this atomic configuration does share some similar aspects. For instance, the distance between the guest atom and Si framework atoms is 3.4–3.75 Å in ternary type I clathrate. Previous density functional theory (DFT) calculations^22^, ^23^ also showed that it is energetically feasible for multiple Li atoms to reside inside clathrate cages and form polyhedral groupings, although these calculations were performed for type II clathrate.

In short, from these NMR results, we can conclude that the framework structure of the clathrate does not change upon lithiation. The absence of lithium silicide peaks in the ^29^Si NMR spectrum of the lithiated type I clathrate studied here is in agreement with the lithiation mechanism being distinct from those observed in the lithiation of other silicon materials such as c‐Si, a‐Si, and SiO. Although the exact nature of the local Li environment is not known, it does show a ^7^Li resonance similar to that observed in other lithium silicides. Recent studies^47^, ^52^ have also shown that ^7^Li chemical shifts in lithium silicides can be observed over a broader range of frequencies when performing measurements at lower temperatures and using 2D NMR, a topic of future study that can better understand the Li local environment in the lithiated ternary type I clathrate.

### 2.4 Postcycling XRD Characterization

**Figure 5:**
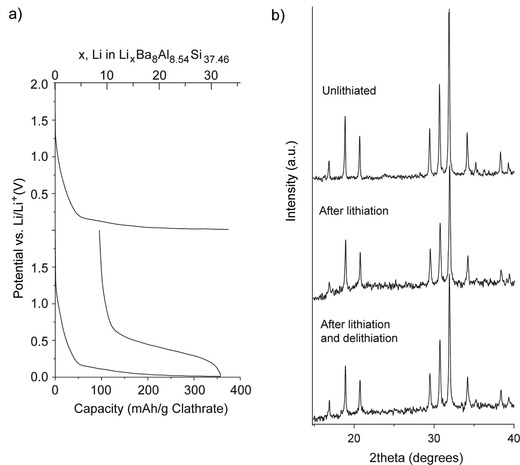
a) Voltage profile of vacuum arc‐melted Ba_8_Al_8.54_Si_37.46_ reaction with Li using galvanostatic cycling at 25 mA g^−1^. b) XRD of electrode before and after cycling.

To confirm the NMR results that the silicon clathrate structures did not change after lithiation, XRD was performed on the vacuum arc‐melted clathrate electrodes after the end of the first charge and after a full lithiation/delithiation cycle. As shown in the voltage profile in **Figure** **5**a, about 31 Li were inserted in the charge, and about 23 Li were extracted in the delithiated electrode. Reflections from the Cu substrate were visible in the XRD patterns (Figure S10, Supporting Information) and the Cu (111) peak was used to align the patterns for comparison. The XRD pattern for the clathrate remained unchanged and no new peaks were observed due to other phases. Notably, there were also no peaks observed associated with formation of c‐Li_15_Si_4_, despite charging <50 mV versus Li/Li^+^ and no significant peak shifts are observed. In other studies, practically guest‐free type II clathrate Na_1.3_Si_136_ 24 and filled Na_24_Si_136_ 23 were observed to transform into c‐Li_15_Si_4_ upon lithiation <50 mV, much like in c‐Si and a‐Si. The absence of c‐Li_15_Si_4_ peaks in the XRD patterns of the lithiated type I clathrate studied here is in agreement with the discharge features that are distinct from delithiation of c‐Li_15_Si_4_. XRD after extended galvanostatic cycling was also performed. The clathrate features in the XRD and dQ plots were still visible in electrodes after 40 cycles at 25 mA g^−1^ and 100 cycles at 100 mA g^−1^ (Figure S7c–f, Supporting Information), suggesting that the clathrate did not undergo any phase transformations even after extended cycling.

**Figure 6:**
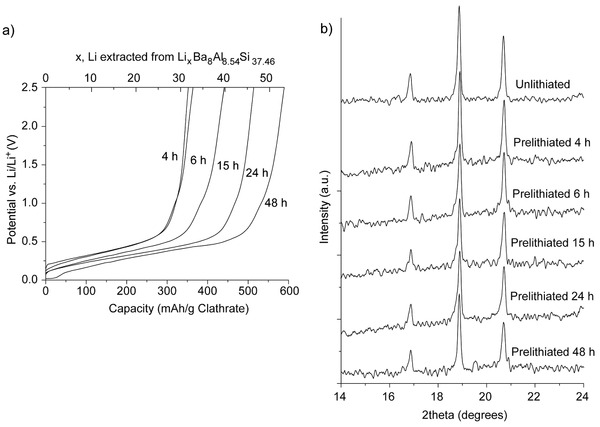
a) Electrochemical delithiation voltage curves and b) XRD patterns after chemical prelithiation with Li metal for different times.

To confirm that the observed electrochemical features were from Li insertion/extraction from the clathrates, chemical lithiation was performed followed by electrochemical delithiation. This prelithiation procedure was performed on vacuum arc‐melted Ba_8_Al_8.54_Si_37.46_ electrodes wetted with Li electrolyte for different periods of time. As shown in **Figure** **6**a, longer prelithiation times corresponded to higher discharge capacities. Slightly higher amounts of Li were extracted from these cells than were observed when using electrochemical lithiation. For instance, 53 Li were extracted after prelithiation for 48 h. The discharge curves obtained after chemical lithiation showed similar sloped profiles as those obtained after electrochemical lithiation. The XRD patterns of the cells after prelithiation are shown in Figure 6b focusing on the first three visible reflections corresponding to the (002), (012), and (112) planes in type I clathrate. The XRD patterns for the full range are shown in Figure S10 (Supporting Information). Importantly, no change in lattice constant was observed, even after insertion of 53 Li. Thus, these results suggest that type I silicon clathrates can be lithiated with little to no volume change. Even though this capacity is about a third of the theoretical capacity for c‐Si, lithiation of c‐Si to a similar state of charge results in approximately 100% volume change. Further cycling of the prelithiated electrodes (Figure S11, Supporting Information) gave electrochemical features associated with the clathrate structure similar to those observed with electrochemical lithiation (Figure 2).

### 2.5 Electrochemical Cycling in Different Electrolytes

**Figure 7:**
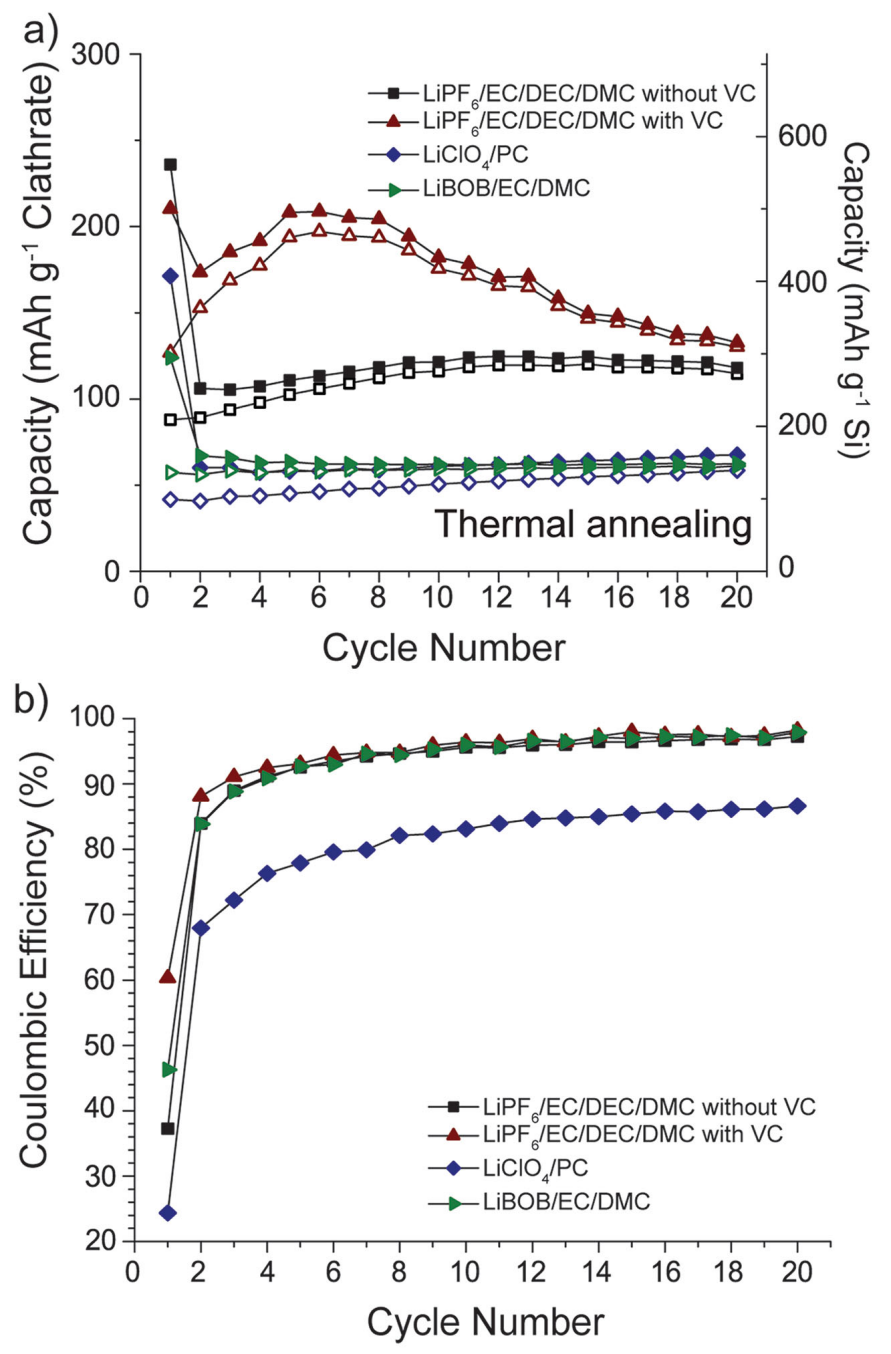
Galvanostatic cycling data for thermally annealed Ba_8_Al_10.39_Si_35.61_ in four different electrolytes using a current density of 25 mA g^−1^. a) Capacity versus cycle life. Capacity calculated using mass of entire clathrate (left axis) or mass of only Si (right axis). b) Coulombic efficiency versus cycle life.

To better understand the origins of the low CE observed in the first few cycles and limited capacity retention when cycling the clathrate electrodes at low current densities, the electrode–electrolyte interphase was further investigated by performing galvanostatic cycling of the thermally annealed clathrate (which displayed the lowest capacities) in different electrolytes. The baseline electrolyte was 1 m LiPF_6_ in EC/DMC/DEC (4:3:3 by volume) with <5% vinylene carbonate (VC) as additive (Figure 3a). We also used 1 m LiPF_6_ in EC/DMC/DEC (1:1:1 by volume), 1 m LiClO_4_ in PC, and 0.7 m lithium bis(oxalato)­borate (LiBOB) in EC/DMC (1:1 by volume). **Figure** **7** compares the electrochemical properties of the clathrate electrode in four different electrolytes using 25 mA g^−1^ and Figure S12 (Supporting Information) shows the voltage profiles and dQ plots. The SEI formation voltages were slightly different in the four different electrolytes. Cycling the clathrate in the LiPF_6_ electrolyte with VC resulted in the highest capacities and CE during all 20 cycles. Comparing the dQ plots in Figure 2b with Figure S12b (Supporting Information), we can see that with the presence of VC in the electrolyte, the SEI layer formed at potentials >0.2 V versus Li/Li^+^ in the first charge, whereas without VC there was a notable peak at 0.6 V versus Li/Li^+^. The samples cycled in LiClO_4_ and LiBOB electrolytes had significantly more SEI layer formation and much lower capacities. These results suggest that the SEI film formed on the clathrate electrode in LiPF_6_ electrolyte with VC additive is more stable than that formed in the other three electrolytes.

### 2.6 Postcycling SEM Imaging

To understand the impact of lithiation on the morphology of the electrode, SEM imaging was performed on the Ar arc‐melted clathrate electrode after the first charge in LiPF_6_ electrolyte with VC additive. Despite the superior cycling in the VC containing electrolyte, SEM imaging showed the electrode is covered by a relatively thick SEI layer (Figure S13, Supporting Information), suggesting the SEI layer may play a large role in the cycling performance of the different clathrates. The electrodes were also examined after washing away the SEI with DMC. Figure S14 (Supporting Information) shows the SEM images and energy‐dispersive X‐ray spectroscopy (EDS) mapping for Ba, Si, and Al signals for Ar arc‐melted clathrate before and after lithiation. The morphologies of the cycled clathrate particles were very similar to the pristine ones. The distribution of the elements was similarly uniform in both images, suggesting that phase segregation of Ba and/or Al did not occur during electrochemical cycling. There was also no evidence of Ba plating on the Li metal counter electrode, as determined by performing EDS on the Li counter electrodes after cycling.

### 2.7 XPS Characterization After Cycling

**Figure 8:**
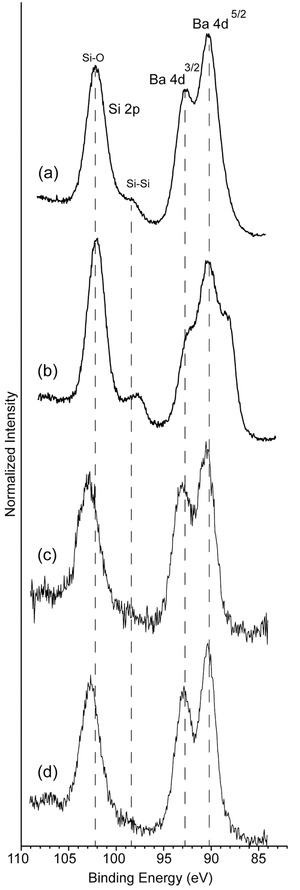
High resolution XPS spectra of thermally annealed Ba_8_Al_10.39_Si_35.61_; a) powder; b) as prepared electrode; c) after lithiation, and d) after one lithiation/delithiation cycle in 1 m LiClO_4_/PC.

XPS was also performed on the clathrate electrodes before and after lithiation. **Figure** **8** shows the Si 2p and Ba 4d high resolution scans for the thermally annealed clathrate Ba_8_Al_10.39_Si_35.61_. The Ba 4d^3/2^ and Ba 4d^5/2^ peaks for the fresh powder are observed at 92.80 and 90.25 eV, respectively (Figure 8a). After electrode preparation, which involved dispersing the clathrate powder into an aqueous slurry with carbon black and carboxymethyl cellulose (CMC) binder followed by coating onto Cu foil and heating, the Ba peaks shifted to slightly lower binding energies and a shoulder appeared at lower binding energies (Figure 8b), which is consistent with the formation of Ba oxides that have lower binding energies than metallic Ba.^55^, ^56^, ^57^ The Si 2p unresolved doublet is centered at about 98.5 eV, corresponding to the Si in the clathrate framework. Compared with regular c‐Si, which has a binding energy at about 99.5 eV for Si–Si, the shift of the Si 2p peak to lower binding energies is possibly due to the interaction of the clathrate Si with Ba and Al, which are more electropositive. These results are also consistent with the XPS results observed for Na_8_Si_46_ clathrate.^58^ The signals attributed to surface Si atoms bound to oxygen centered at approximately 102.5 eV are higher than the Si–Si signal, suggesting the presence of a native oxide of a few nms. As reported previously, Si atoms can form various suboxides via bonding to different numbers of oxygen atoms at the Si–SiO_2_ interface.^58^, ^59^, ^60^ The Si 2s core level also supports the presence of metallic Si and Si oxides. In contrast to the 2p core level, which consists of a spin‐orbit coupled doublet, the Si 2s core level is composed of singlet that is easier to fit. A tentative fit further supports the presence of Si^0^ intermediate oxides and Si^4+^ oxide species (Figure S15a, Supporting Information). XPS was also performed on electrodes after the end of the first charge and after a full lithiation/delithiation cycle. The electrode was charged in 1 m LiClO_4_/PC at 25 mA g^−1^, with a lithiation capacity of 183 mAh g^−1^ corresponding to insertion of about 16 Li (Figure S15b, Supporting Information). For this sample, the XPS spectrum (Figure 8c) was relatively unchanged except the Si^0^ signal at 98.5 eV was no longer visible due to surface coverage by the SEI layer. The spectrum of the lithiated sample also showed contributions between 103–105 eV from Si bonded to SEI species.^61^ The XPS spectrum (Figure 8d) for the electrode that was lithiated and then delithiated (Figure S15c, Supporting Information) looked very similar, which indicates that the SEI layer was not removed during oxidation.

## 3 Conclusion

In conclusion, we have demonstrated, for the first time, the electrochemical insertion of Li into type I silicon clathrates based on Ba_8_Al*_y_*Si_46−*y*_. According to the electrochemical analysis, the lithiation/delithiation processes are proposed to occur in single phase, solid solution‐type reactions. Our experimental results show no discernible structural or volume changes even after insertion of large amounts of Li (up to 44 electrochemically, or 53 chemically) into the clathrate structure. Postcycling analysis with NMR and XRD did not show evidence of the transformation of the clathrate into other crystalline phases and the electrochemical characteristics did not indicate transformation into amorphous silicon or lithium silicides. The observed properties are in stark contrast to lithiation of c‐Si and a‐Si, which become amorphous and undergo much larger volume changes. These results suggest that silicon clathrates could be very promising candidates as durable anodes for lithium‐ion batteries.

The CE observed <80% when cycling at low rates or using long prelithiation times indicate that there may be problems with poorly passivated surfaces that are unable to stop side reactions related to electrolyte decomposition. This may result in a thick SEI layer that affects the interfacial reaction kinetics. This has also been seen in silicon anodes, particularly those composed of nanostructures.^10^, ^62^ The distorted tetrahedral arrangement of the silicon atoms in clathrate structures may result in an enhanced reactivity with the electrolyte, but this needs to be further investigated. While the high molecular weight of the Ba guest atoms, which are required for formation of the clathrate structure, limits the gravimetric energy density of this electrode, future work into synthesis and characterization of other silicon clathrate compositions may lead to anode candidates with more attractive specific capacities.

## 4 Experimental Section

*Synthesis of Silicon Clathrates*: Polycrystalline powders were synthesized using thermal annealing and arc‐melting from the elements using nominal starting compositions of Ba_8_Al_8_Si_38_. Thermal annealing was performed by reaction of Ba (Sigma‐Aldrich 99% pieces under oil), Al foil, and Si powder (−325 mesh and 99.99% trace metals basis). The mineral oil was removed from Ba by washing with hexanes followed by toluene. Stoichiometric amounts of components totaling about 2 g in weight were mixed together and compressed into pellets with approximately 5 MPa of pressure in a hydraulic press. These pellets were then sealed in tantalum foil and heated in a tube furnace under 5 CFPS argon flow. The samples were heated to 1100 °C over 6 h, held at this temperature for 6 h, then ramped down to room temperature over 6 h. Arc melting in an Ar‐filled glovebox was performed on similarly prepared pellets. The clathrate was also synthesized in bulk (200 g) from admixtures of thoroughly mixed Ba, Al, and Si powders using large‐volume vacuum arc‐melting (Sophisticated Alloys, Inc., Butler, PA).

To remove any BaSi_2_ side product and unreacted c‐Si from the product, a three‐step procedure was performed after the synthesis. First, the synthesized product was reduced into smaller particle sizes about 1–10 μm by ball‐milling for 40 min in a SPEX 8000 mill. Second, the powder was suspended in de‐ionized water and treated with 3 m HCl for 12 h to remove the BaSi_2_.^20^, ^28^, ^29^ After treatment, the powder was recovered using vacuum filtration and washed with de‐ionized water. Finally, the powder was treated with 1 m NaOH for 12 h to remove c‐Si,^20^, ^28^, ^29^ then recovered using vacuum filtration and washed with de‐ionized water and dried. Previous studies have shown that silicon clathrates and their guest ions are insensitive to moisture, water, and acids other than hydrofluoric acid (HF)^15^ and the alkaline treatment did not appear to affect the clathrate.

*Chemical Prelithiation*: Inside an Ar‐purifed glovebox, clathrate samples were prelithiated^39^ by wetting clathrate electrodes with Li electrolyte (1 m LiPF_6_ in EC/DMC/DEC with VC) and then pressing them against Li foil. The samples were placed in polybags and a weight was placed on top to ensure uniform contact between the clathrate and the lithium. Prelithiation was carried out for different times (4, 6, 15, 24, and 48 h). Then, the Li metal was removed and the samples were assembled into half‐cells and electrochemically delithiated to determine the extent of Li insertion during prelithiation. XRD analysis was performed on a separate set of samples of similar film thickness and prelithiated for the same times as mentioned before using the same procedures. For chemical lithiation of samples for NMR characterization, samples were lithiated for 48 h at 100 °C without the use of Li electrolyte.

*WDS Characterization*: Wavelength dispersive X‐ray spectroscopy (WDS, JEOL JXA‐8530F) was used to determine the composition of the silicon clathrate after postsynthesis etching. The clathrate powder was mixed with epoxy and cured overnight at room temperature in a chemical hood. Then the sample was ground and polished to a flat surface for subsequent analyses. Elemental Si, Al, and barite were used as standards to determine the net elemental intensities for Si, Al, and Ba. 10 different locations were randomly chosen and analyzed for each sample.

*NMR Characterization*: Prior to the static solid‐state NMR measurements, the samples were flame‐sealed in 3 mm NMR tubes to prevent exposure to oxygen and moisture over the course of the experiments. The ^29^Si static NMR measurements were conducted on a Varian 400 MHz VNMRS Wide‐Bore spectrometer equipped with a 3.2 mm triple resonance HXY probe configured for ^1^H^29^Si double resonance. The spectra were collected using the Hahn echo sequence with a 3.5 μs π/2 pulse, a tau delay of 50 μs, a recycle delay of 150 ms, 393k transients, an acquisition time of 1 ms, with no proton decoupling. Additional ^29^Si spectra were collected for the lithiated Ar arc‐melted clathrate samples to determine if any appreciable amount of lithium silicide had been generated during lithiation. These spectra were collected with a recycle delay of 5 s, and 12k transients. The ^29^Si spectra were indirectly referenced to tetramethylsilane (TMS) in the solid state by using TTSS. The ^7^Li static NMR spectra were obtained using a single pulse experiment with a 3.5 μs π/2 pulse, a recycle delay of 15s, and 512 transients. The ^7^Li spectrum was referenced with respect to a 1 m LiCl solution.

*XRD Characterization*: Powder XRD characterization of films was performed using monochromatic CuKa radiation (*λ* = 1.5405Å) (Panalytical X'pert Pro). Lithiated films were protected from air exposure with Kapton films. Rietveld analysis was performed using the Bruker‐AXS Topas 4.2 or PANalytical X'Pert HighScore Plus software. The data were refined for the space group Pm$\bar{3}$n (No. 223) using a Split PearsonVII peak type and background fit to a seventh‐order polynomial function. For the vacuum arc‐melted sample, the analysis yielded a = 10.48942(50) Å, *R*_p_ = 11.53%, *R*_wp_ = 15.10%, *R*_exp_ = 13.55%, *R*_Bragg_ = 1.867, and χ^2^ = 1.12. For the Ar arc‐melted sample, the analysis yielded a = 10.5097(20) Å, *R*_p_ = 11.54%, *R*_wp_ = 14.17%, *R*_exp_ = 11.34%, *R*_Bragg_ = 2.831, and χ^2^ = 1.25. For the thermally annealed sample, the analysis yielded a = 10.560(11) Å, *R*_p_ = 13.88%, *R*_wp_ = 17.79%, *R*_exp_ = 13.61%, *R*_Bragg_ = 3.770, and χ^2^ = 1.31.

*SEM Characterization*: The clathrate samples were imaged using a FEI XL30 scanning electron microscope (SEM). For postmortem analysis of the lithiated clathrate, anhydrous dimethyl carbonate (DMC, Sigma‐Aldrich) was used to wash away excess electrolyte after removal of the electrode from the cell. To further remove the SEI layer, the electrode was soaked in DMC for 24 h.

*XPS Characterization*: XPS was performed using a PHI 3056 XPS spectrometer with Al Kα radiation (1486.6 eV) at a measurement pressure below 10^−8^ Torr. The energy scale calibration of the instrument is checked regularly using Ag and Au standards. The samples were disassembled in an Ar‐purified glovebox and rinsed with anhydrous DMC and transferred under vacuum directly to the XPS sample chamber using an air‐tight transfer device. High resolution scans were acquired at 350 W with 23.5 eV pass energy and 0.05 eV energy step. Survey scans were measured at 350 W with 93.9 eV pass energy and 0.3 eV energy step. The energy scale of the spectra was calibrated to the C 1s peak at 284.5 eV.

*Electrochemical Characterization*: Slurries were prepared by mixing the clathrate sample with 10 wt% carbon black, 10 wt% polyvinylidene difluoride (PVDF) in N‐methyl pyrrolidone (NMP) or 10 wt% carboxymethyl cellulose (CMC) in de‐ionized water as solvent. No significant difference in cycling performance was observed using these two binders. The results presented here were obtained by using PVdF as binder for vacuum arc‐melted clathrate and CMC as binder for thermally annealed and Ar arc‐melted clathrates. Electrodes were prepared by coating the slurries onto Cu foil current collectors using a Meyer rod followed by heating at 120 °C to remove the solvent. The thickness of the films was approximately 25 μm. Half‐cells were assembled using Li metal as the counter electrode. Unless otherwise noted, the electrolyte used was 1 m LiPF_6_ in ethylene carbonate (EC): dimethyl carbonate (DMC): diethylcarbonate (DEC) mixture (4:3:3 by volume) with vinylene carbonate (VC) additive (MTI). The cells were assembled inside an Ar‐filled glovebox and sealed in aluminized polyethylene laminate bags.

Electrochemical testing was performed using a Biologic VMP3 galvanostat/potentiostat. Potentiodynamic cycling with galvanostatic acceleration (PCGA) was used to perform electrochemical potential spectroscopy^63^ using a 5 mV potential step amplitude with a threshold current of 25 mA g^−1^ of clathrate over the voltage range of 2.5–0.01 V versus Li/Li^+^. Galvanostatic measurements were performed over the same voltage range using a current rate of 5, 25, 50, or 100 mA g^−1^ of clathrate.

## Supporting information

As a service to our authors and readers, this journal provides supporting information supplied by the authors. Such materials are peer reviewed and may be re‐organized for online delivery, but are not copy‐edited or typeset. Technical support issues arising from supporting information (other than missing files) should be addressed to the authors.

Supplementary
