# Supplementary material for: Type I Clathrates as Novel Silicon Anodes: An Electrochemical and Structural Investigation
Source: Adv Sci (Weinh). 2015 May 5;2(6):1500057. doi: 10.1002/advs.201500057 (PMC5115401; doi:10.1002/advs.201500057)
Supplement: Supplementary file 1 — Supplementary [file ADVS-2-0d-s001.pdf]

Copyright WILEY-VCH Verlag GmbH & Co. KGaA, 69469 Weinheim, Germany, 2015.

ADVANCED SCIENCE
Open Access

Supporting Information

for *Adv. Sci.*, DOI: 10.1002/advs.201500057

Type I Clathrates as Novel Silicon Anodes: An Electrochemical and Structural Investigation

*Ying Li, Rahul Raghavan, Nicholas A. Wagner, Stephen K. Davidowski, Loïc Baggetto, Ran Zhao, Qian Cheng, Jeffery L. Yarger, Gabriel M. Veith, Carol Ellis-Terrell, Michael A. Miller, Kwai S. Chan, and Candace K. Chan**

Copyright WILEY-VCH Verlag GmbH & Co. KGaA, 69469 Weinheim, Germany, 2013.

Supporting Information

Type I Clathrates as Novel Silicon Anodes: An Electrochemical and Structural Investigation

*Ying Li, Rahul Raghavan, Nicholas A. Wagner, Stephen Davidowski, Loïc Baggetto, Ran Zhao, Qian Cheng, Jeffery L. Yarger, Gabriel M. Veith, Carol Ellis-Terrell, Michael A. Miller, Kwai S. Chan, Candace K. Chan**

Supporting Figures S1-S15

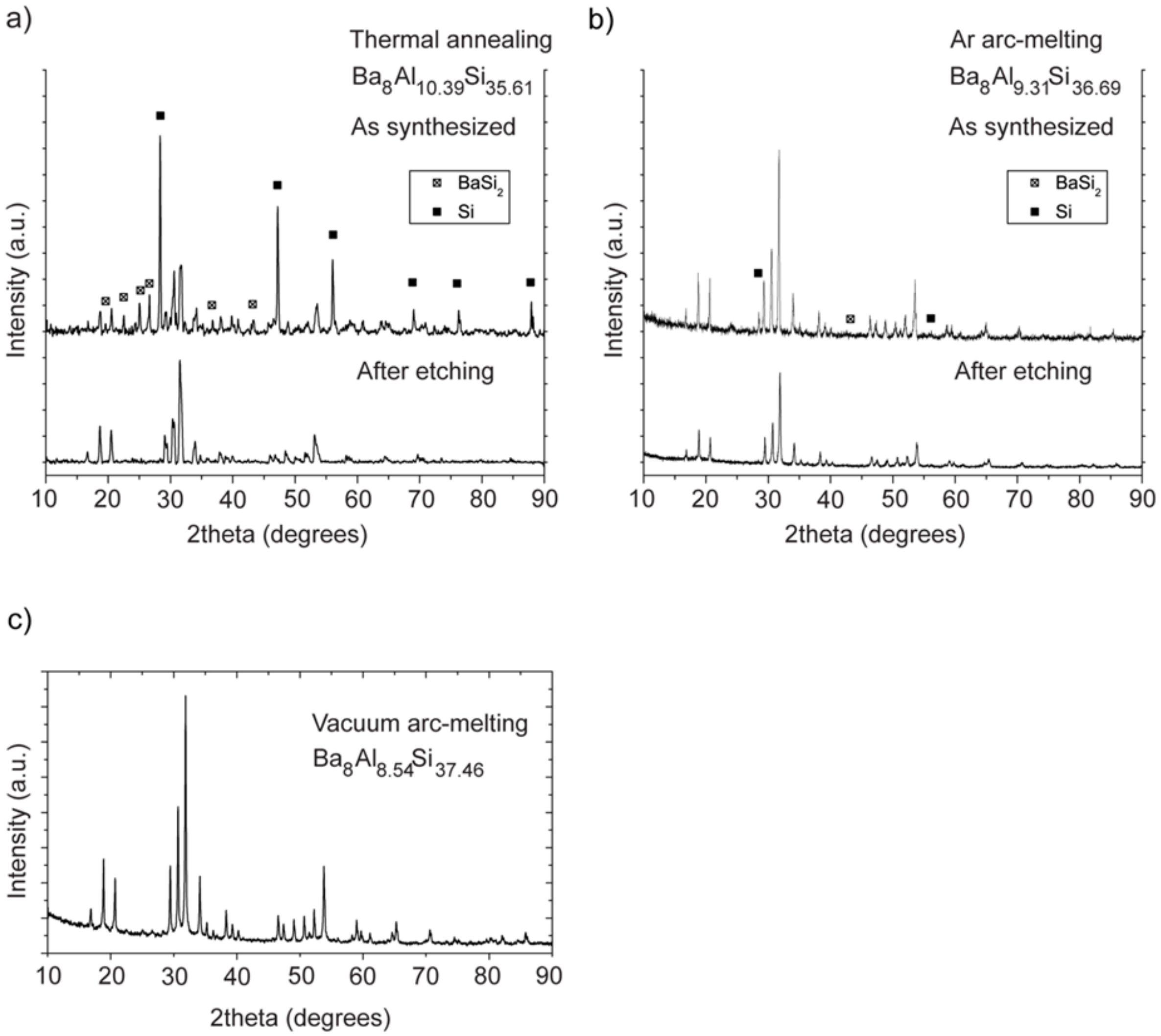

Figure S1. Powder XRD patterns **(a)** As-made $Ba_8Al_{10.39}Si_{35.61}$ prepared by thermal annealing (top) and after HCl and NaOH etching (bottom). **(b)** As-made $Ba_8Al_{9.31}Si_{36.69}$ prepared by Ar arc-melting (top) and after HCl and NaOH etching (bottom). **(c)** As-made $Ba_8Al_{8.54}Si_{37.46}$ prepared by vacuum arc-melting. The compositions of the clathrate samples were determined using the average compositions obtained from WDS.

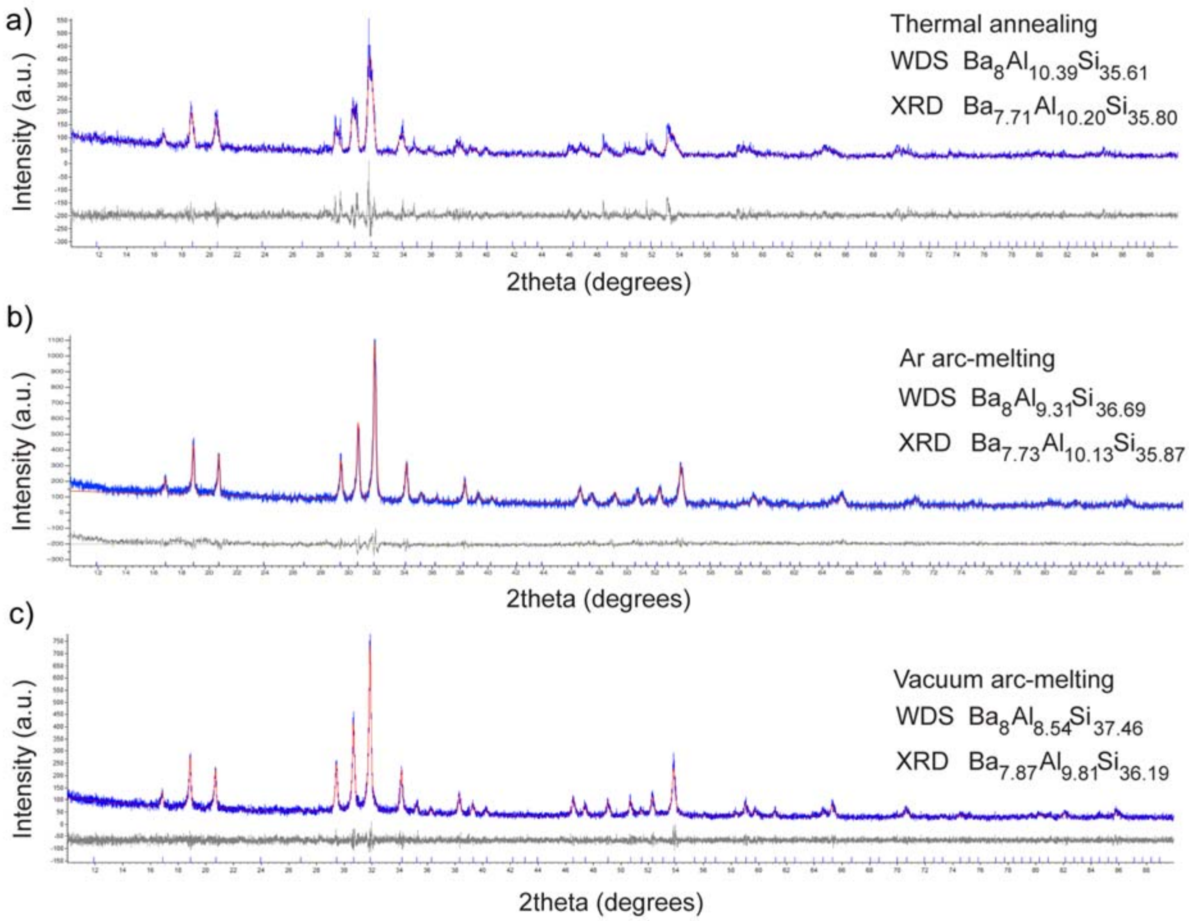

Figure S2. Rietveld refinement results for **(a)** thermal annealed, **(b)** Ar arc-melted, and **(c)** vacuum arc-melted clathrate. Blue traces are the experimental patterns, red traces are the calculated patterns, and grey traces are the residuals. Compositions obtained from the refinement are compared with those obtained from WDS.

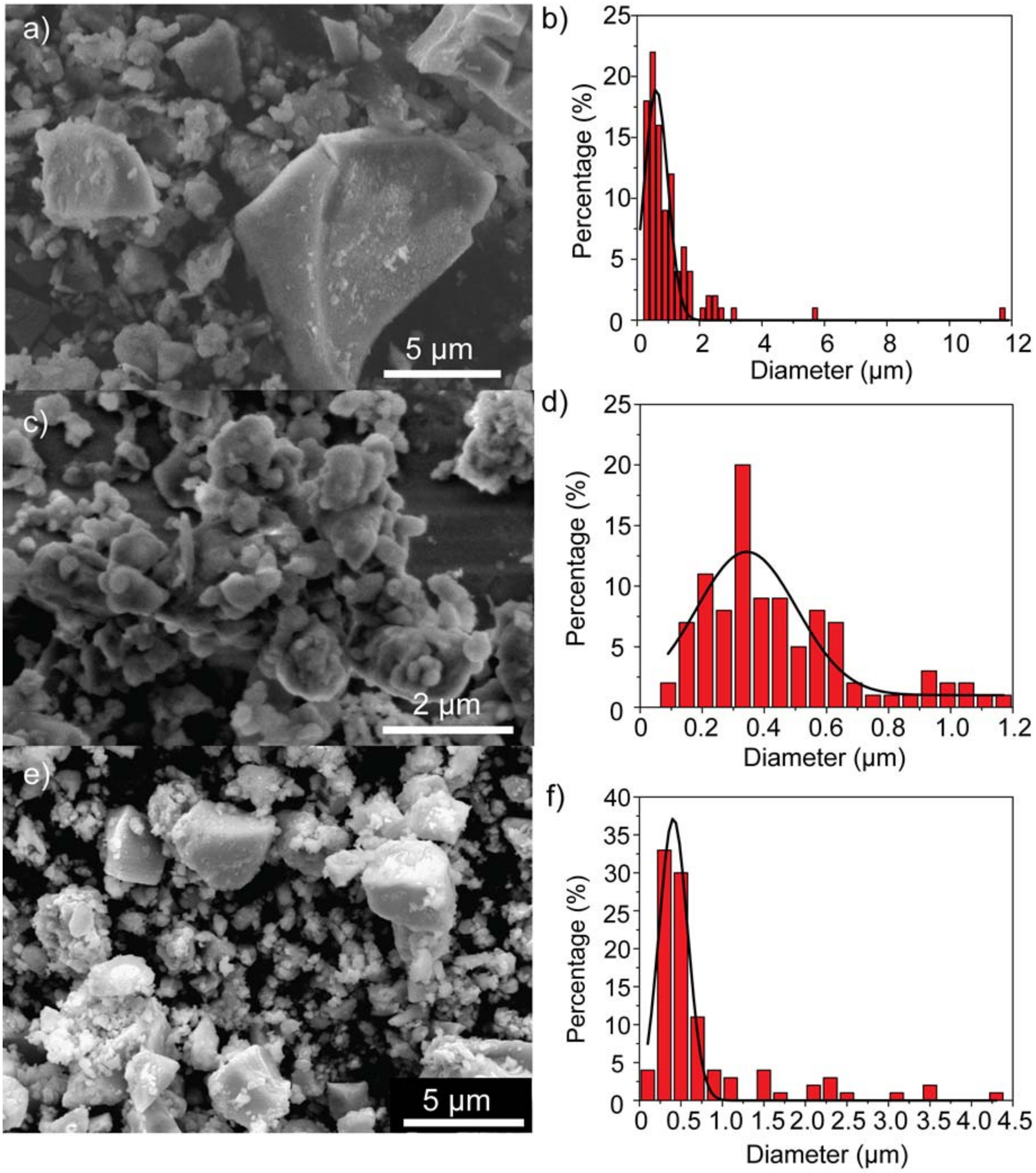

Figure S3. SEM images and particle size distribution of clathrate after ball-milling. Material synthesized using **(a,b)** thermal annealing, **(c,d)** Ar arc-melting, and **(e,f)** vacuum arc-melting. Gaussian peak fitting showed peaks at 0.57, 0.34, and 0.41 μm for the thermal annealed, Ar arc-melted, and vacuum arc-melted samples, respectively.

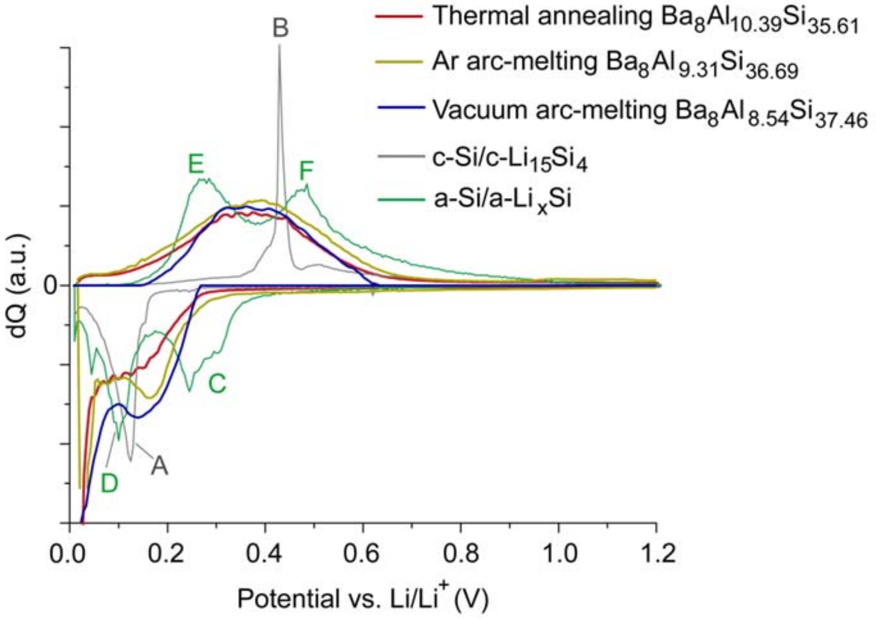

Figure S4. Differential charge plots of c-Si and a-Si compared to those for the second cycles of the clathrates. The y-axis has been scaled to aid comparison between the samples. The labels correspond to the following lithiation and delithiation processes in c-Si and a-Si reported in reference [1]:

A. Two-phase reaction between c-Si and a-Li_xSi at 0.125 V vs. Li/Li^+ during lithiation. Below 50 mV vs. Li/Li^+, the a-Li_xSi transforms to c-$Li_{15}Si_4$.

B. Two-phase reaction between c-$Li_{15}Si_4$ and delithiated a-Si at 0.43 V vs. Li/Li^+ during discharge.

C. Single-phase (e.g. solid solution) reaction of Li into a-Si.

D. Single-phase reaction of Li into a-Li_xSi.

E. Single-phase delithiation reaction with Li removal from Li-rich a-Li_xSi.

F. Single-phase delithiation reaction with Li removal from Li-poor a-Li_xSi.

[1]M. N. Obrovac, L. Christensen, *Electrochem. Solid-State Lett.* **2004**, *7*, A93.

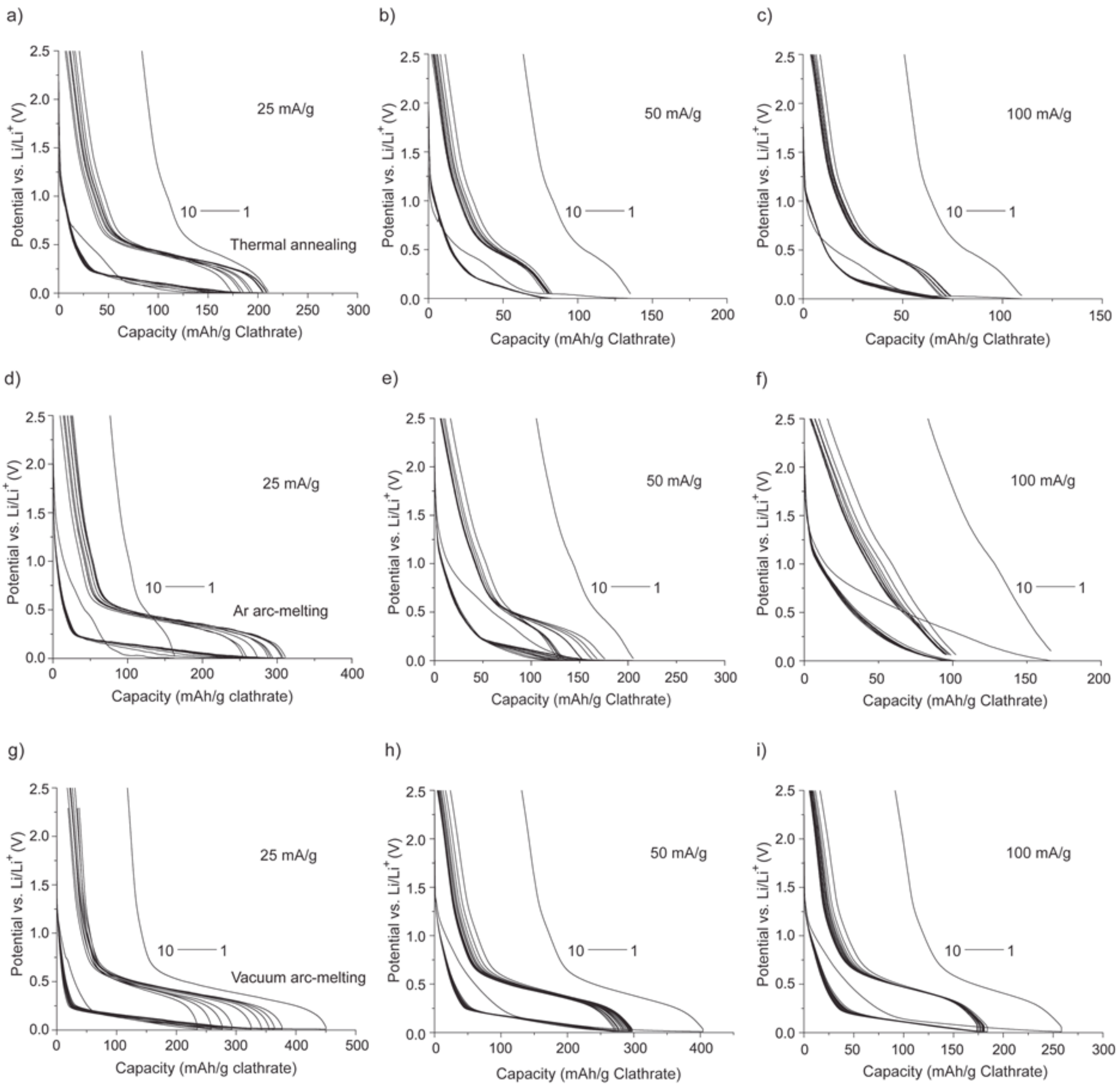

Figure S5. Galvanostatic cycling data for **(a,b,c)** thermal annealed $Ba_8Al_{10.39}Si_{35.61}$, **(d,e,f)** Ar arc-melted $Ba_8Al_{9.31}Si_{36.69}$, and **(g,h,i)** vacuum arc-melted $Ba_8Al_{8.54}Si_{37.46}$ using different current rates at 25 mA g^{-1}, 50 mA g^{-1}, and 100 mA g^{-1}.

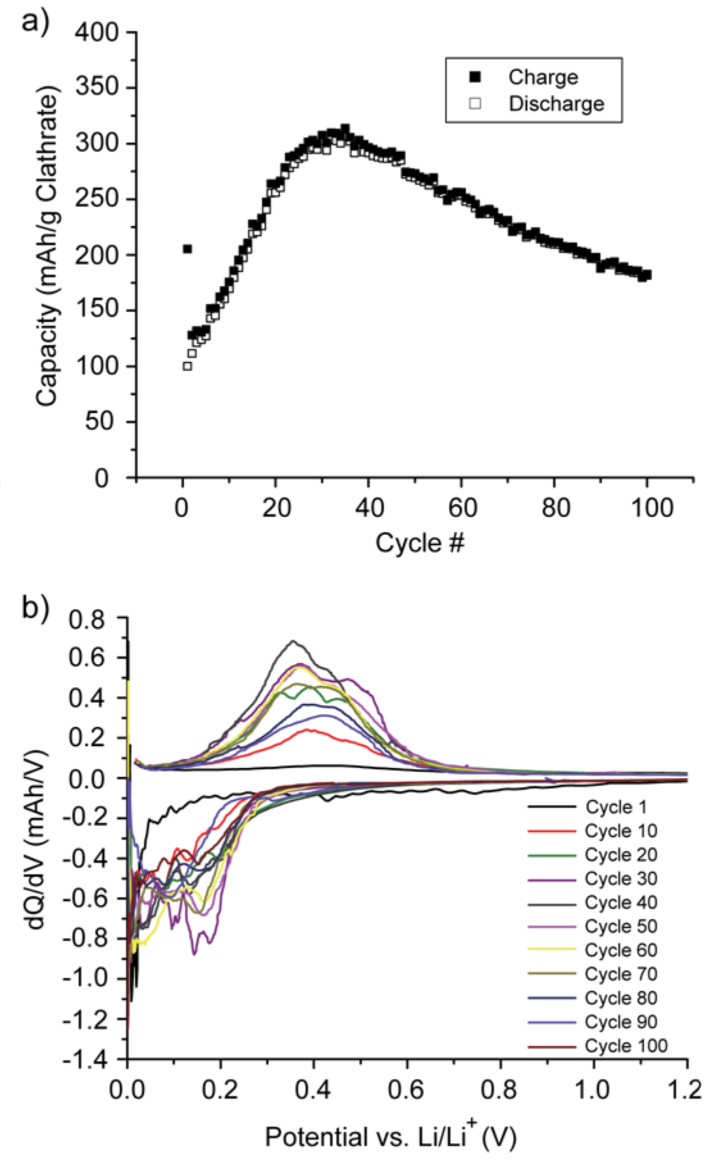

Figure S6. Galvanostatic cycling of Ar arc-melted $Ba_8Al_{9.31}Si_{36.69}$ using 50 mA g^{-1} **(a)** Capacity vs. cycle life, **(b)** dQ/dV vs. V plots, every 10th cycle shown.

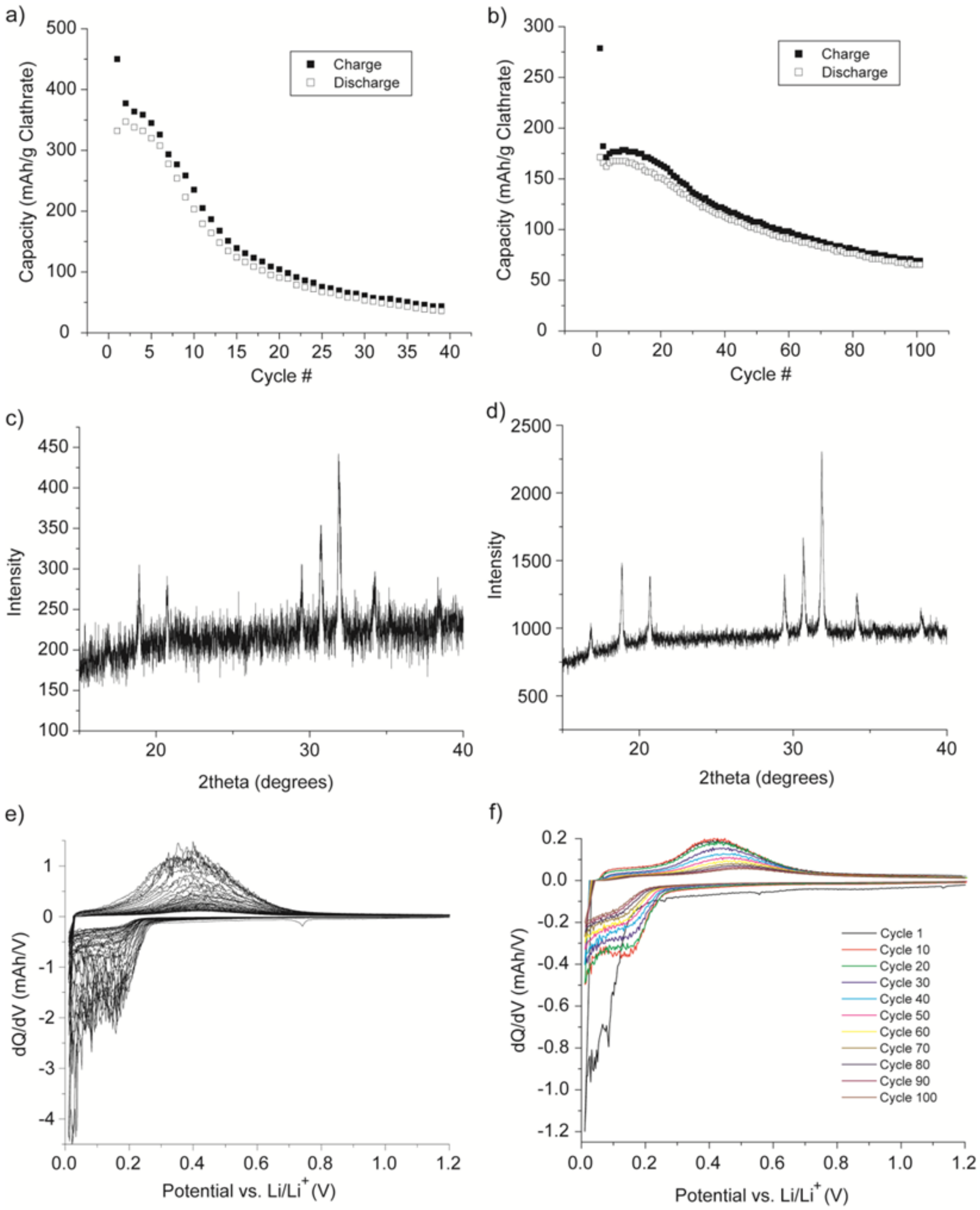

Figure S7. Galvanostatic cycling of vacuum arc-melted $Ba_8Al_{8.54}Si_{37.46}$ using **(a,c,e)** 25 mA g^{-1} and **(b,d,f)** 100 mA g^{-1}. **(a,b)** Capacity vs. cycle life using mass of clathrate; **(c,d)** XRD of sample after extended cycling; **(e,f)** dQ/dV vs. V plots, **(e)** shows all 40 cycles while **(f)** shows every 10th cycle.

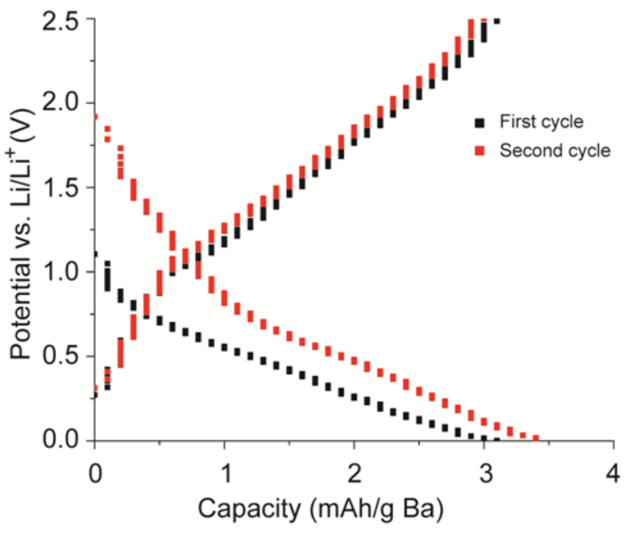

Figure S8. Galvanostatic cycling of Ba metal in Li half-cells using 25 mA g^{-1}.

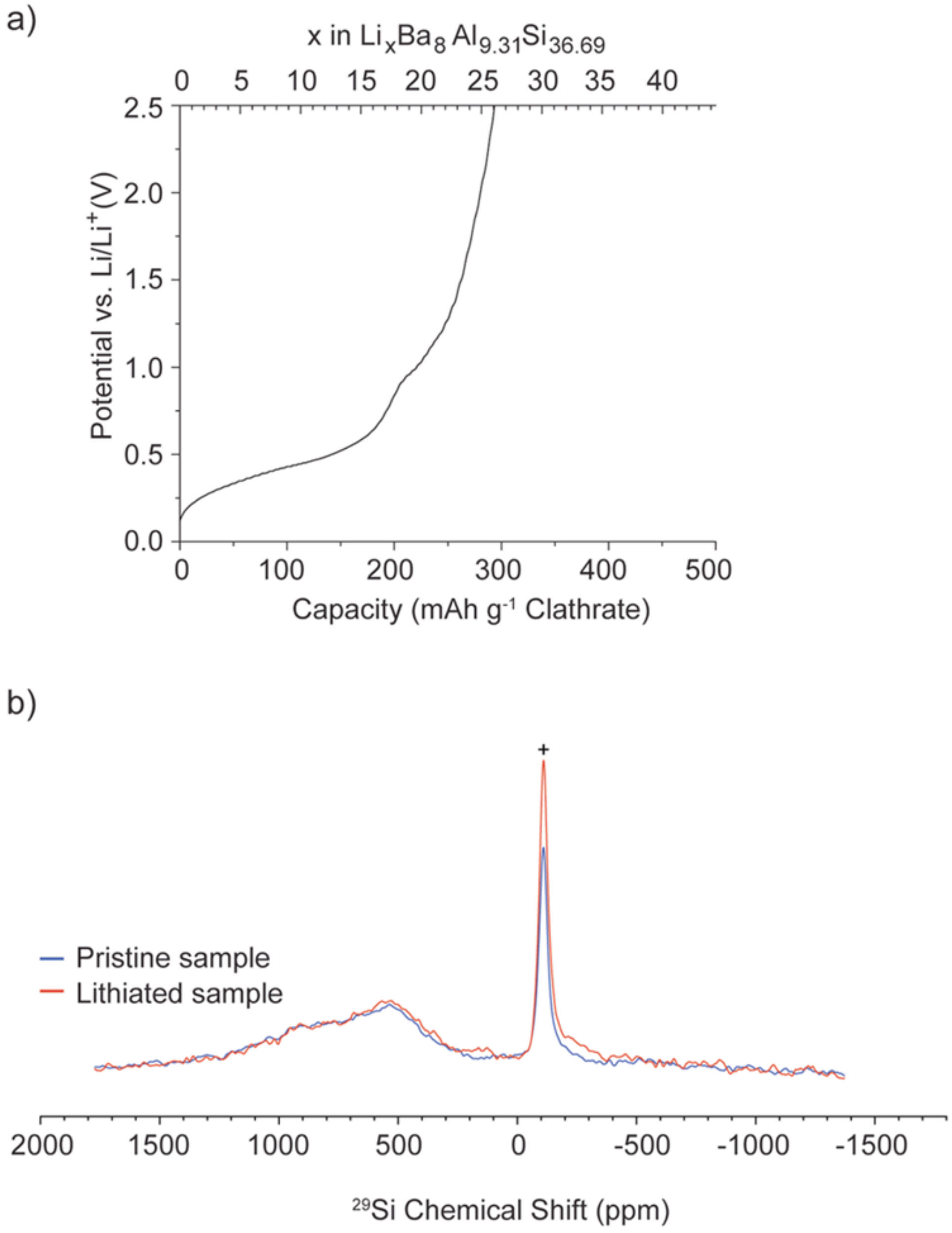

Figure S9. **(a)** Electrochemical delithiation voltage curve of Ar arc-melted $Ba_8Al_{9.31}Si_{36.69}$ after chemical lithiation for 48 h at 100 °C. **(b)** ^{29}Si static NMR spectra of Ar arc-melted $Ba_8Al_{9.31}Si_{36.69}$ with 5 s relaxation delay. Peak labeled with "+" is from the SiO_2 glass tube used in static NMR.

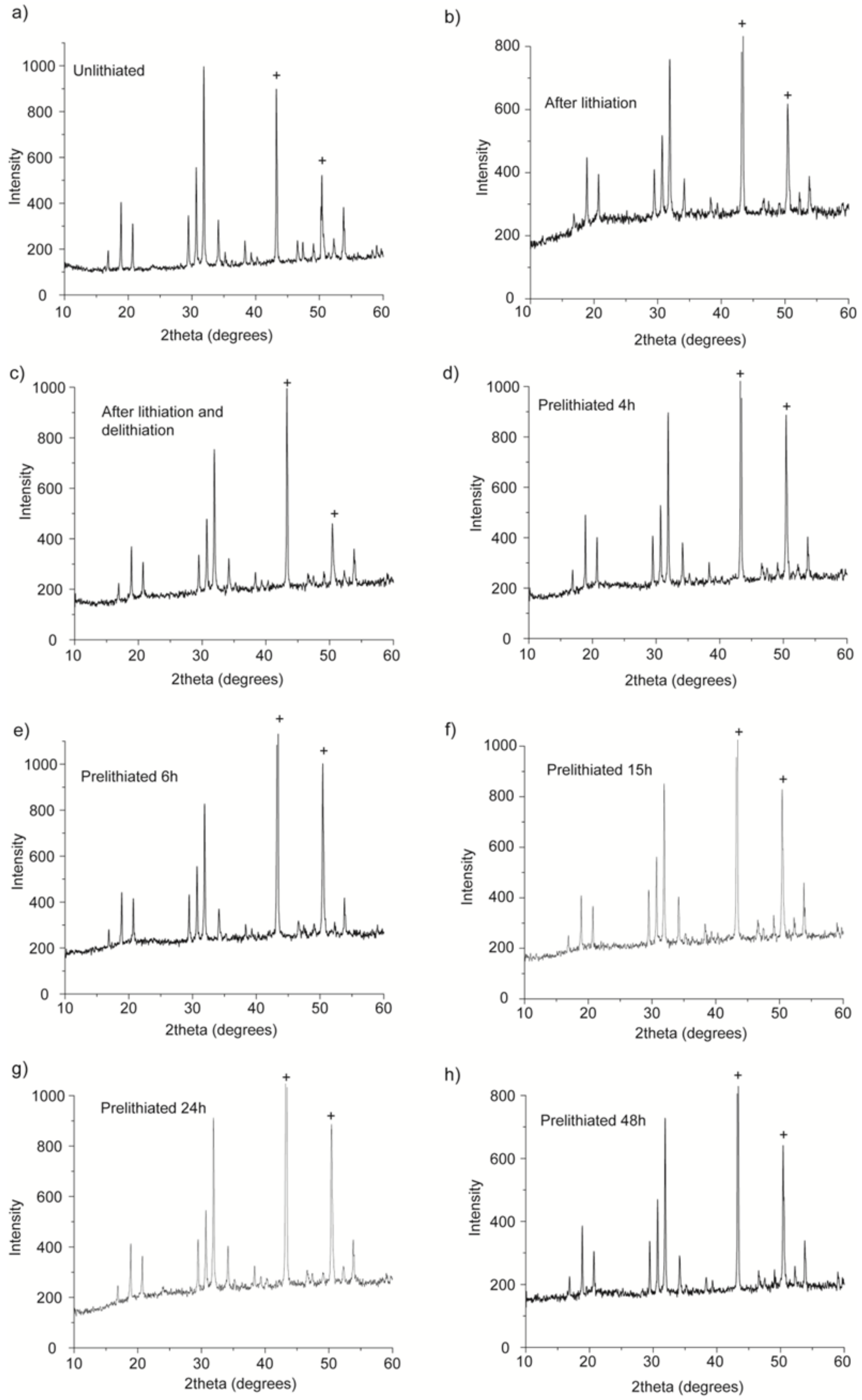

Figure S10. Full XRD scans of vacuum arc-melted $Ba_8Al_{8.54}Si_{37.46}$ films on Cu foil. Peaks labeled with + are from Cu. **(a)** unlithiated, **(b)** after first charge, **(c)** after one lithiation/delithiation cycle, **(d) – (h)** after prelithiation for various time periods between 4 – 48 h.

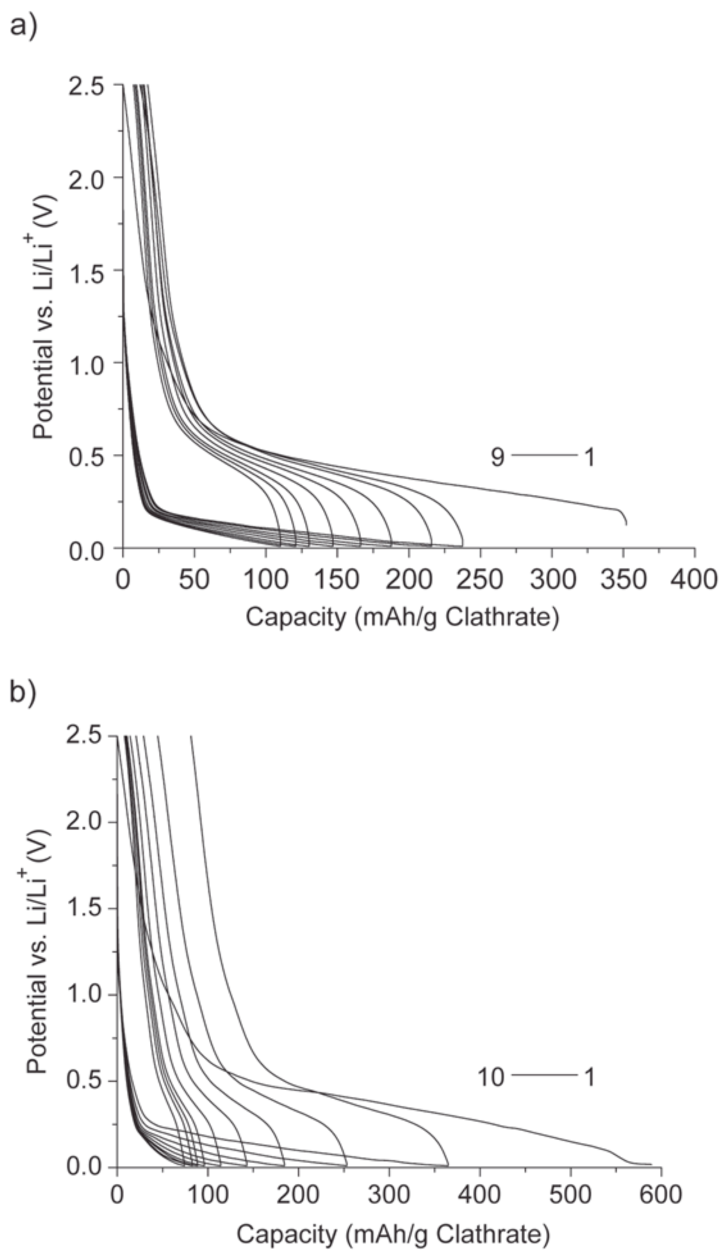

Figure S11. Galvanostatic voltage profiles obtained using 25 mA g^{-1} for vacuum arc-melted $Ba_8Al_{8.54}Si_{37.46}$ electrodes prelithiated for **(a)** 4 hours, **(b)** 48 hours.

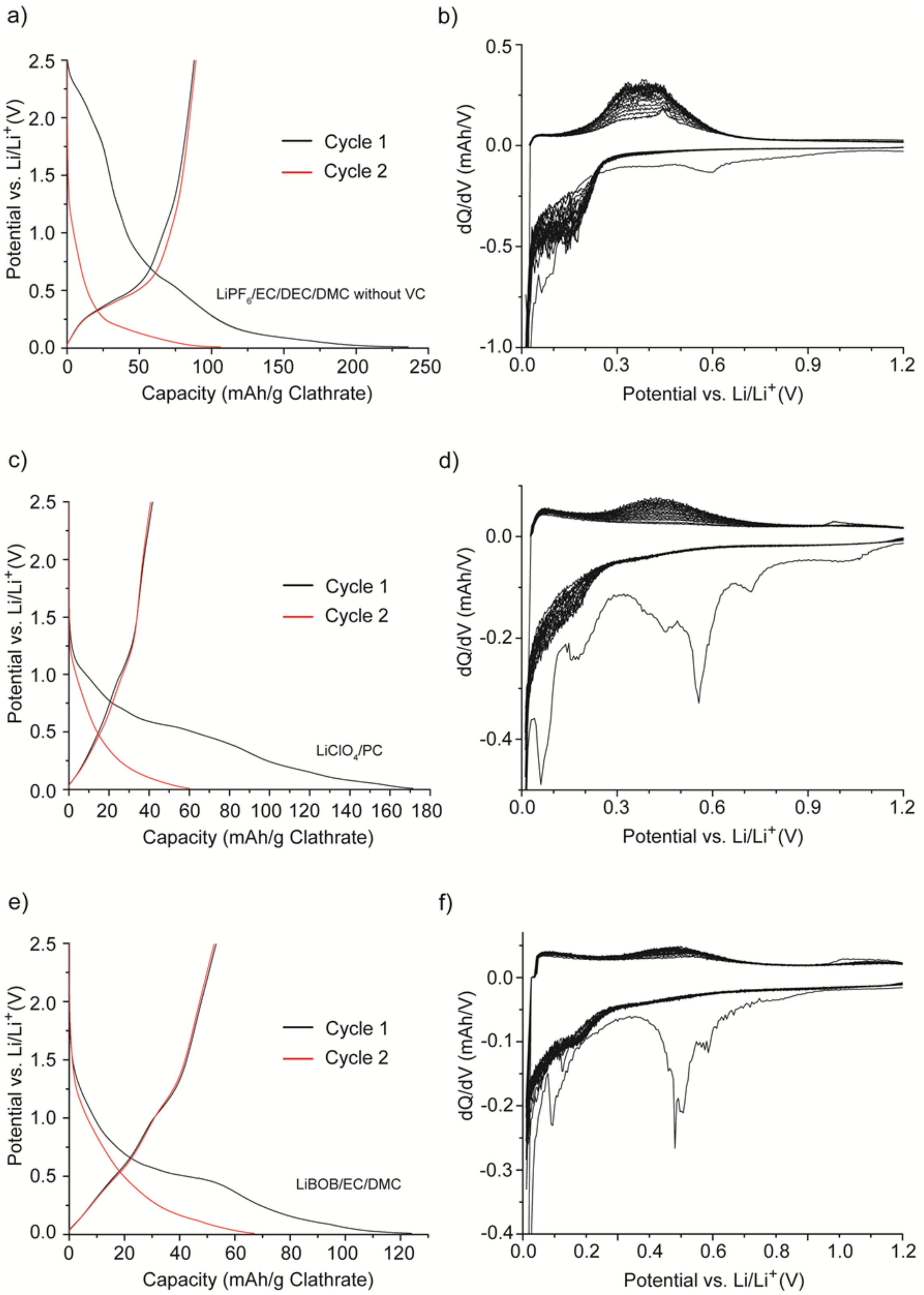

Figure S12. (a,c,e) Voltage profiles and **(b,d,f)** differential charge plots from galvanostatic cycling of thermal annealed clathrate in different electrolytes. **(a,b)** 1 M $LiPF_6$ in EC/DMC/DEC (1:1:1 by volume); **(c,d)** 1 M $LiClO_4$ in PC, **(e,f)** 0.7 M LiBOB in EC/DMC (1:1 by volume).

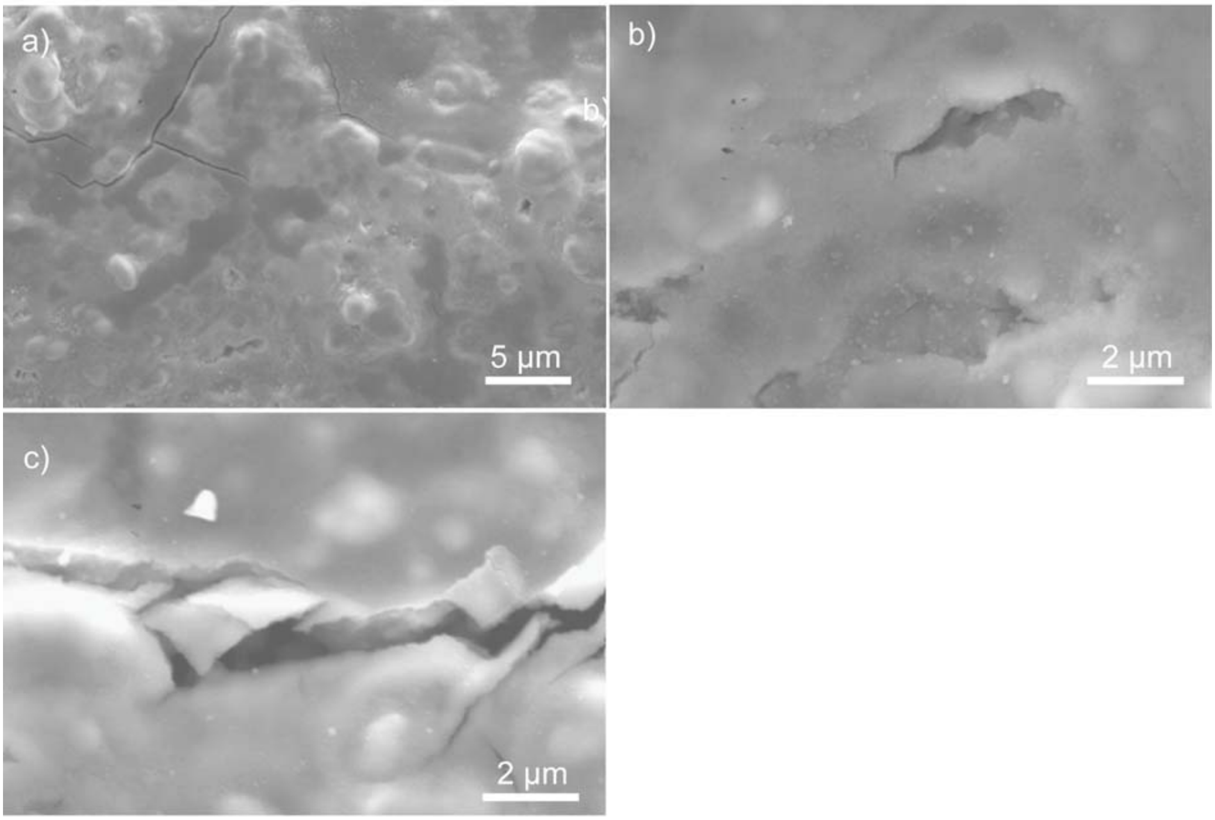

Figure S13. SEM images of Ar arc-melted $Ba_8Al_{9.31}Si_{36.69}$ clathrate electrode in different locations after first lithiation in 1 M $LiPF_6$ electrolyte in EC/DMC/DEC (4:3:3) with VC additive.

a)

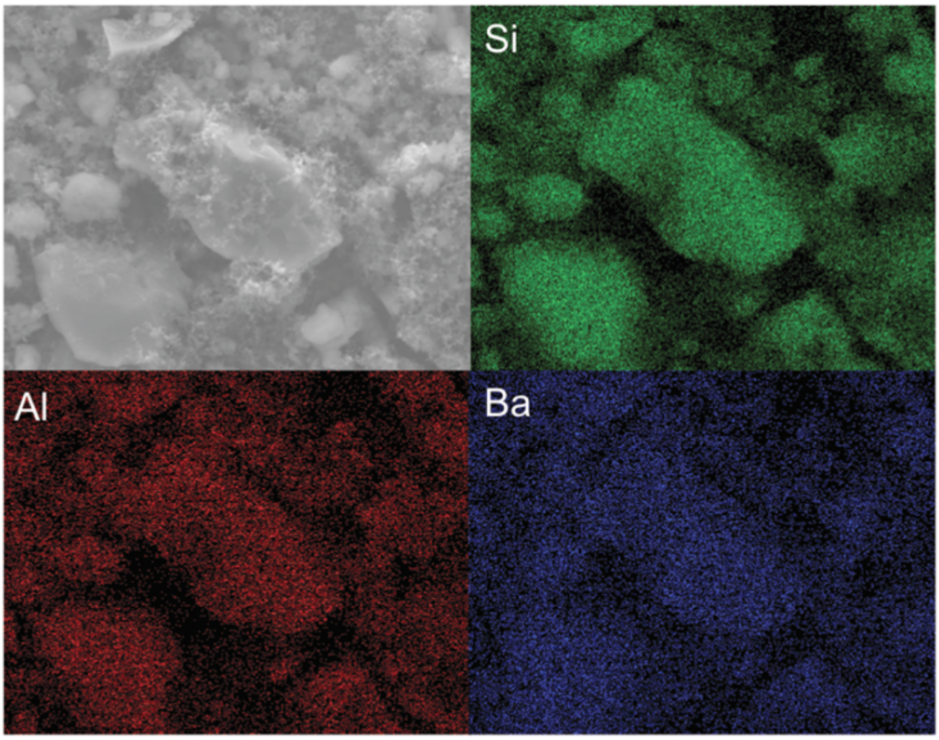

b)

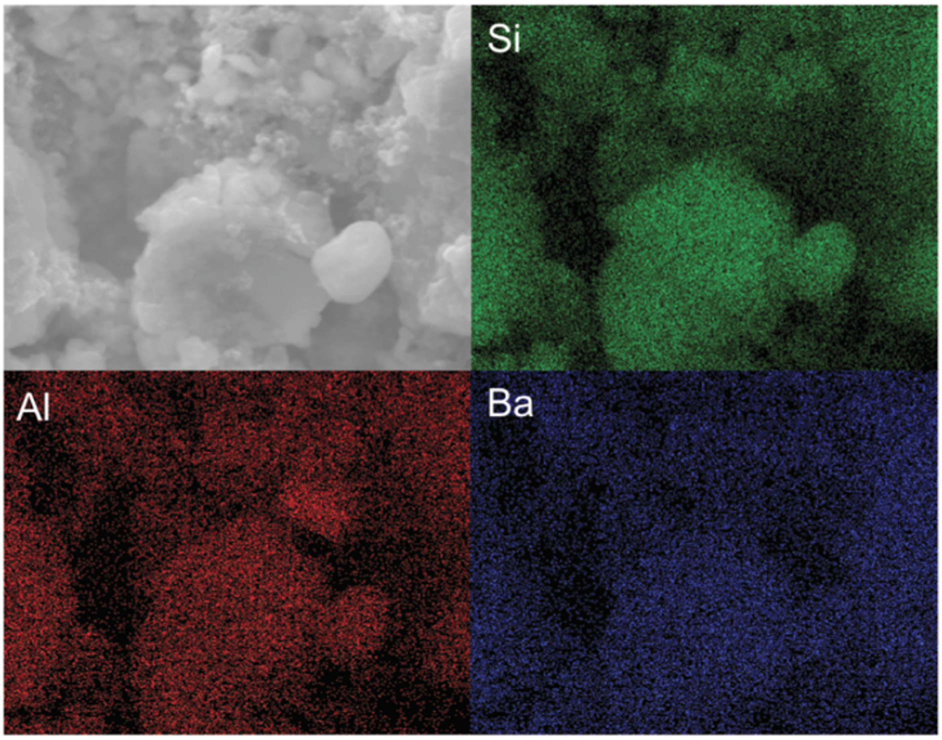

Figure S14. EDX mapping of Ar arc-melted $Ba_8Al_{9.31}Si_{36.69}$ clathrate (a) before and (b) after one charge. The electrolyte (1 M $LiPF_6$ in EC/DC/DMC with VC) and SEI were removed by soaking the electrode in DMC for 24 hours followed by washing.

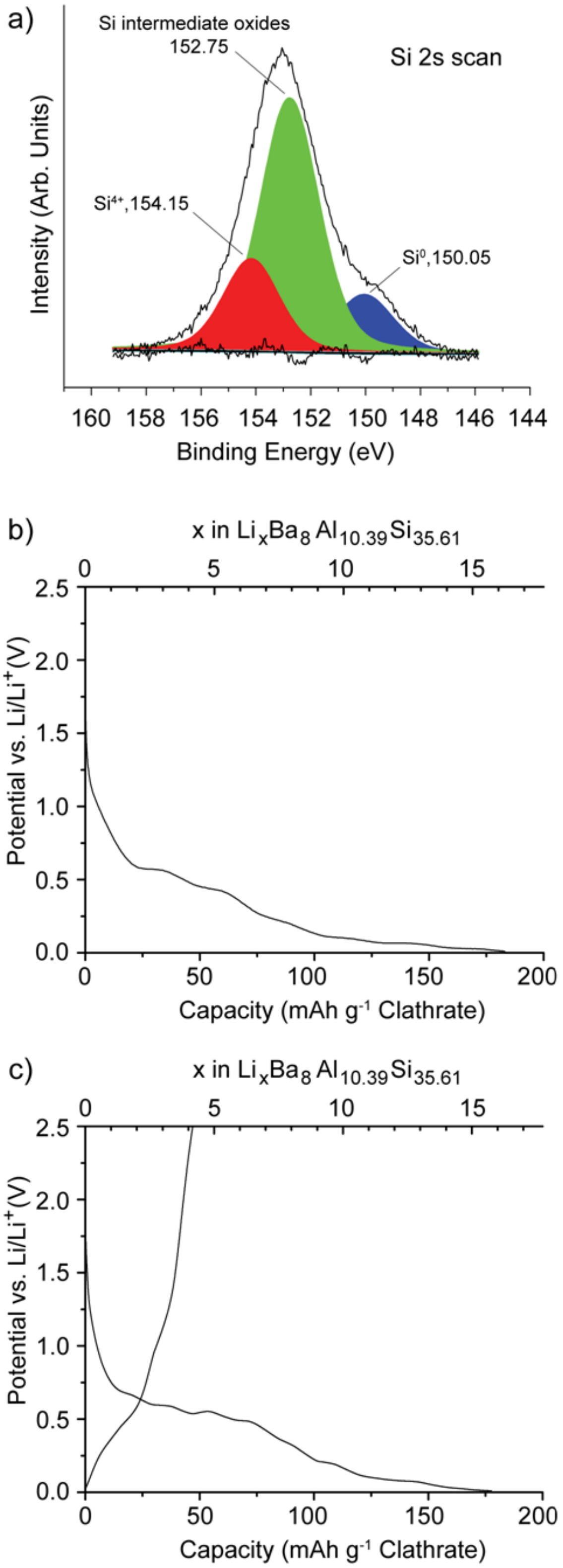

Figure S15. (a) Fitted Si 2s spectrum for the fresh thermal annealed $Ba_8Al_{10.39}Si_{35.61}$ clathrate powder. (b) Voltage profile after the end of the first charge and (c) Voltage profile after a full lithiation/delithiation cycle of thermal annealed $Ba_8Al_{10.39}Si_{35.61}$ using galvanostatic cycling at 25 mA g^{-1} in 1 M $LiClO_4$/PC.
